# Structural Modifications on Chalcone Framework for Developing New Class of Cholinesterase Inhibitors

**DOI:** 10.3390/ijms23063121

**Published:** 2022-03-14

**Authors:** Ginson George, Vishal Payyalot Koyiparambath, Sunitha Sukumaran, Aathira Sujathan Nair, Leena K. Pappachan, Abdullah G. Al-Sehemi, Hoon Kim, Bijo Mathew

**Affiliations:** 1 Department of Pharmaceutical Chemistry, Amrita School of Pharmacy, AIMS Health Sciences Campus, Amrita Vishwa Vidyapeetham, Kochi 682 041, India; ginsongeorge239@gmail.com (G.G.); vishalpk11@gmail.com (V.P.K.); sunithasukumaran27@gmail.com (S.S.); sujuaathira@gmail.com (A.S.N.); leenakpappachen@aims.amrita.edu (L.K.P.); 2 Research Center for Advanced Materials Science (RCAMS), King Khalid University, P.O. Box 9004, Abha 61413, Saudi Arabia; agsehemi@kku.edu.sa; 3 Department of Pharmacy, Sunchon National University, Suncheon 57922, Korea; 4 Research Institute of Life Pharmaceutical Sciences, Sunchon National University, Suncheon 57922, Korea

**Keywords:** chalcones, acetylcholinesterase, butyrylcholinesterase, structure activity relationships

## Abstract

Due to the multifaceted pharmacological activities of chalcones, these scaffolds have been considered one of the most privileged frameworks in the drug discovery process. Structurally, chalcones are α, β-unsaturated carbonyl functionalities with two aryl or heteroaryl units. Amongst the numerous pharmacological activities explored for chalcone derivatives, the development of novel chalcone analogs for the treatment of Alzheimer’s disease (AD) is among the research topics of most interest. Chalcones possess numerous advantages, such as smaller molecular size, opportunities for further structural modification thereby altering the physicochemical properties, cost-effectiveness, and convenient synthetic methodology. The present review highlights the recent evidence of chalcones as a privileged structure in AD drug development processes. Different classes of chalcone-derived analogs are summarized for the easy understanding of the previously reported analogs as well as the importance of certain functionalities in exhibiting cholinesterase inhibition. In this way, this review will shed light on the medicinal chemistry fraternity for the design and development of novel promising chalcone candidates for the treatment of AD.

## 1. Introduction

A family of cholinesterase (ChE) catalyzes the hydrolysis of the neurotransmitter acetylcholine (ACh) into acetic acid and choline. Acetylcholinesterase (AChE) is strategically located at all cholinergic sites and serves to inactivate ACh simultaneously, while butyrylcholinesterase (BChE), also known as pseudocholine esterase, present in plasma, serves to metabolize ingested esters. The main difference between AChE and BChE is that AChE inhibition is more sensitive to physostigmine, while BChE inhibition is more sensitive to organophosphates. The main role of AChE is the termination of ACh action, while the function of BChE is the hydrolysis of ingested esters [1,2].

The cholinergic enzyme AChE is predominantly present at postsynaptic neuromuscular junctions, particularly in muscles and nerves. The main function of AChE is to inhibit ACh dispersal and activation of adjacent receptors by terminating neuronal transmission and signaling between synapses. AChE is mainly distributed in nervous tissues such as the cerebellum, autonomic nervous systems, peripheral nervous systems, and brainstem. Red blood cell membranes and skeletal muscles also contain AChE [3].

AChE catalyzes the breakdown, hydrolysis, and inactivation of ACh, thereby controlling its amount in the synapse. AChE is a serine hydrolase; through acid-based reactions, a tetrahedral intermediate is formed. The formation of a new acylated serine is facilitated via histidine, which helps in the proton transfer between the oxygen molecules within the serine, and ACh, which leads to the removal of the choline group. The protonated histidine is stabilized by the aspartate, and the deacylation of acylated serine takes place. This results in the regeneration of free AChE [4].

AChE has been extensively studied in the tissues involved in Alzheimer’s disease (AD) since the finding of the cholinergic deficiency. AD is one of the most common neurodegenerative disorders, characterized by the diminishing of memory, orientation to physical surroundings, and language. Neurons present in the hippocampus and cortex are degenerated irreversibly as well as progressively. The reduction in the synthesis of ACh is one of the main factors, as per the cholinergic hypothesis. The inhibition of the biological activity of AChE results in the increase of cholinergic levels in the brain. The degradation of ACh is prevented by the AChE inhibitors; thus, ACh concentration is increased, and this can improve the function of neural cells. The concentration of the ACh level elevates at the synaptic junction as a result of AChE inhibition, and also permits the potentiation of the signal. This action eventually decreases the amount of choline absorbed and increases the number of M2 muscarinic receptors.

**Figure 1:**
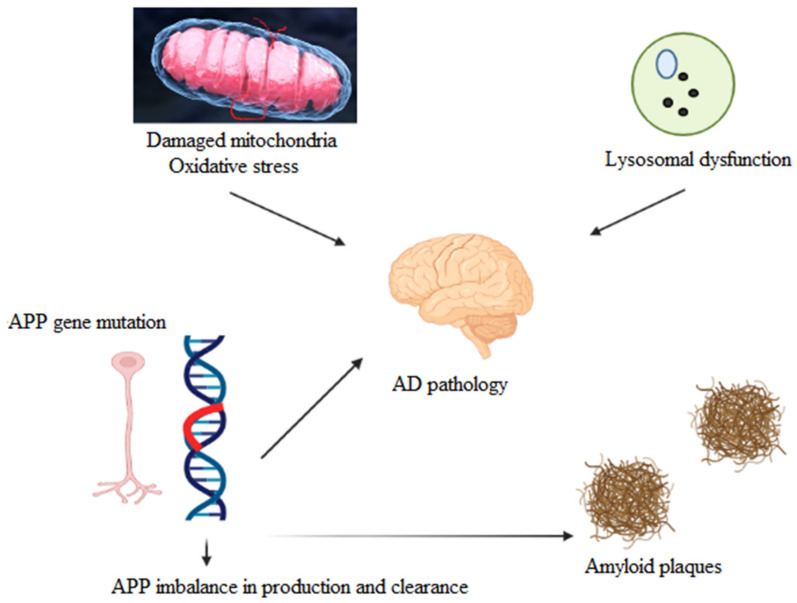
Pathophysiology of Alzheimer’s disease (AD).

Several other critical factors are involved in the pathogenesis of AD (Figure 1), including the progressive synthesis and aggregation of β-amyloid (Aβ), a proteolytic fragment derived from amyloid precursor protein (APP). The role of cholinergic modulation and other functional consequences of AChE inhibition in the processing of APPs may affect neurons and protect them from a variety of neurological insults. Aβ may be deposited into insoluble plaques by interacting directly with AChE [5,6].

According to the crystal structure of AChE, the catalytic triad of serine, histidine, and glutamate is positioned at the bottom of a narrow 20 A˚ deep gorge that penetrates halfway through the enzyme and widens near its base. Aromatic residues line the ligand-binding cavity, accounting for approximately 40% of the cavity surface. Due to its affinity for binding cationic ligands, the peripheral anionic site (PAS) at the gorge’s entrance was originally thought to include multiple negatively charged amino acids. The crystal structure shows that there are not enough acidic amino acids close to the ligand-binding cavity. ACh is assumed to bind to the PAS first, then diffuse down to the catalytic site quickly [7,8].

Numerous crystal structures have been used for molecular modelling studies, as follows: crystal structure of mouse AChE inhibited by non-aged methamidophos (2JGE) [9], recombinant human AChE in complex with donepezil (PDB Code: 4EY7) [10,11], quaternary ligand binding to aromatic residues in the active-site gorge of AChE (PDB Code: 1ACJ) [12], human AChE complexed with fasciculin-II, glycosylated protein (PDB Code: 1B41) [13], and homo sapiens AChE phosphorylated by sarin (PDB Code: 5FPQ) [14].

**Figure 2:**
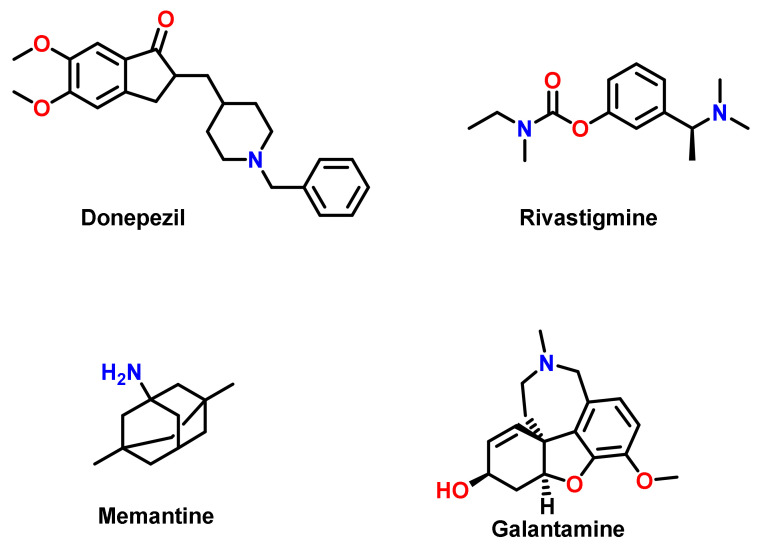
Chemical structures of clinically used drugs for AD.

Acetylcholinesterase inhibitors (AChEIs) are the most commonly prescribed treatment strategy for AD. AChEIs have been found to reduce the progression of the disease as well as improve attention span. These compounds have unique patterns of structural diversity, possessing distinctive specificities for different types of ChE. After the introduction of AChEIs, cholinergic drugs like donepezil, galantamine, and rivastigmine were considered as first-line pharmacotherapy for mild to moderate AD (Figure 2). As ChEs are inhibited by AChEIs or anticholinesterases, the neurotransmitter action is both enhanced and prolonged. Based on the mechanism of action, AChEIs are classified into irreversible and reversible types [15].

Memory, thinking, language, judgment, and other mental processes are treated with reversible AChEI drugs. The level of ACh is upheld by these drugs, which boosts the cholinergic neurotransmission pathway. Donepezil, rivastigmine, and galantamine as reversible AChEIs, and memantine as the NMDA receptor antagonist, are the FDA- and the European Medicines Agency (EMA)-approved drugs to treat the cognitive manifestations of AD. Donepezil binds to the PAS and delays the deposition of amyloid plaque. Rivastigmine inhibits both AChE and BChE by binding at the ecstatic part of the active site. The selective AChEI galantamine interacts with the anionic subsite, as well as with the aromatic gorge, and also at the nicotinic cholinergic receptor. Thus, this molecule acts as an allosteric ligand and induces the modulation [16,17,18,19]. Irreversible AChEIs exert their effects through the non-reversible phosphorylation of esterases in the central nervous system. These are substrate analogs to ACh, and like natural substrate, they enter the active site via covalently binding to the serine–OH group [20].

**Figure 3:**
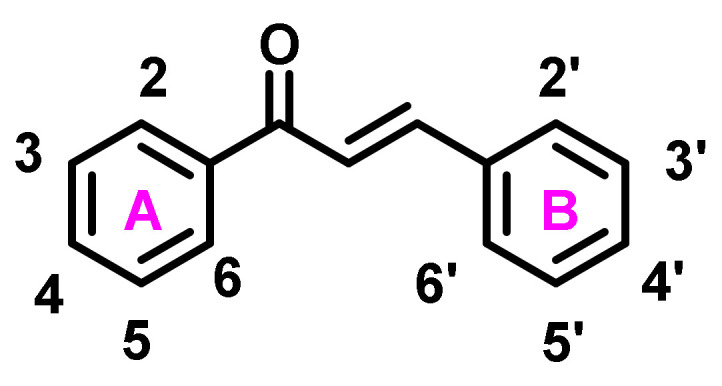
Structure and numbering of chalcone scaffold.

Chalcone (Figure 3) is a simple chemical framework found in a wide range of naturally occurring substances, including fruits, vegetables, and teas. The name “chalcone” comes from the Greek word “chalcos”, which means “bronze”, because most natural chalcones have a bronze tint [21]. The chemical scaffold of chalcone molecules is 1,3-diaryl-2-propen-1-one, also known as chalconoid, that present in trans and cis isomers, with the trans isomer being more stable at high temperatures [22]. The word “chalcone”, as used in several papers, refers to compounds containing an “unsaturated ketone system” [23].

The A ring refers to the phenyl ring linked to the carbonyl group, whereas the B ring refers to the other benzene ring [24]. The presence of α, β unsaturated carbonyl functional group, that acts as a potential Michael acceptor, allows interaction with sulfhydryl of cysteine residue or other thiol groups. Chalcones have been well researched, with several reviews being published. Condensation processes involving basic or acid catalysis are commonly used for the preparation of chalcones. Despite the fact that chalcones are a sort of readily synthesizable and unsaturated ketone, a rising number of innovative approaches, processes, and various catalysts or reaction conditions have lately been described due to their intriguing biological activities [25]. Several bioinspired chalcone derivative syntheses and bioactivity evaluations have been published in the literature due to their structural simplicity and therapeutic promise [26,27,28,29]. The earliest attempts to synthesize chalcones were made in the 1800s and continued over the next two centuries [30]. The Claisen–Schmidt reaction is a method of condensing benzaldehyde and methyl ketone in the presence of catalysts. In an enolate mechanism, the chalcone is produced from the aldol product by dehydration, whereas in an acid catalysis method, it is produced by an enol process [31]. There are many chemical reactions reported for the synthesis of chalcones, including the Suzuki reaction, Heck reaction, Wittig reaction, Julia–Kocienski Olefination, and other cross-coupling reactions [32]. Chalcones have a wide range of biological activities, owing to their compact structures and Michael acceptor characteristics, which make them tolerant of a wide range of biological molecules and allow them to interact with them quickly or reactively [33].

Chalcones are the building blocks of numerous physiologically fascinating chemicals derived from natural sources, and they have piqued researchers’ curiosity for decades. Various biological activities of chalcones, such as anticancer, anti-inflammatory, antituberculosis, antimalarial, antioxidant, antimicrobial activity, antidiabetic, and antiviral activities, and cancer prevention and neuroprotective effects have been reported [34]. This means that there is a wide range of natural chalcones with an innumerable number of biological activities. Some examples of chalcones in nature are cardamonin and curcumin [35]. Fluorescent chalcones have also been discovered to be a promising candidate for studying cellular targets. By changing the polarity of solvents, chalcones with both electron-donating and accepting groups can be utilized to enhance quantum yields via the intermolecular charge transfer (ICT) process [36]. As a result, due to its versatility in skeletal alteration, the chemistry of chalcone-based derivatives remains appealing to researchers. Despite the fact that these substituted chalcones have a variety of medicinal uses and characteristics, their recognition abilities have yet to be investigated. Chalcone serves as both an optical active moiety and a recognition unit in chalcone derivatives, allowing them to selectively detect target analytes [37], mainly focusing on current improvements in employing chalcone as a favored scaffold in medicinal chemistry, with an emphasis on research related to AD therapy as ChEIs.

## 2. Structural Attachment of Various Chemical Functionalities on Chalcone Scaffold (Ring A and B)

The structural modification of chalcones has been explored by numerous reports, wherein substituent-dependent pharmacological activities have resulted. For a promising drug candidate for AD, penetration ability through the blood–brain barrier (BBB) is essential. The structural modifications to the chalcone are also made by the consideration of BBB permeability. Some of these reports utilized the logP value, while in vitro parallel artificial membrane permeation assay of the blood–brain barrier (PAMPA-BBB) was also reported for expressing the BBB permeation. A logP value of less than 2 ± 0.7 is considered to have greater potential to cross the BBB. In the case of the PAMPA assay, a permeability (Pe) greater than 4 × 10^−6^ cm/s is considered a high permeation, while less than 2 × 10^−6^ cm/s is considered a low permeation. For those analogs with Pe in the range of 2 to 4 × 10^−6^ cm/s, the permeation ability is uncertain in nature.

Initial discussions in the present reports mainly focus on the incorporation of various substituents to the chalcone aromatic rings.

### 2.1. Chalcone Derivatives with Various Substituents Attachment

**Figure 4:**
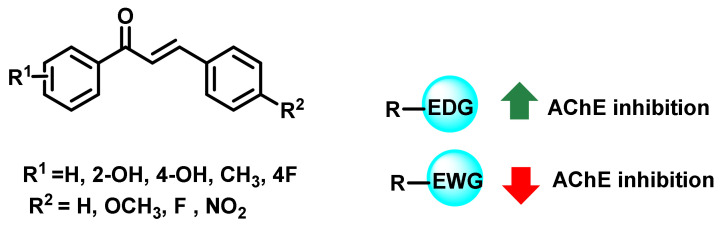
Chalcone derivatives with simple substitutions by Aslan et al. [38].

Aslan et al., reported the AChE inhibitory properties of numerous chalcone analogs with various electron-donating and withdrawing substituents (Figure 4) [38]. The reported analogs exerted varied activity in the range of K_i_ values from 0.61 to 86.11 μM. Further, these analogs exerted a competitive mode of enzyme inhibition. The attachment of electron-donating groups (EDG) resulted in an enhanced AChE inhibitory activity, while electron-withdrawing groups (EWG) resulted in a decremented activity [38].

**Figure 5:**
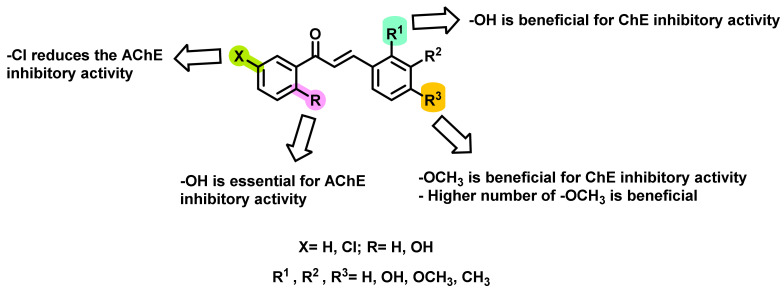
Chalcone derivatives with simple substitutions by Hasan et al. [39].

In another study, the effects of substituents such as hydroxy, methoxy, and chloro groups substituted in the chalcone scaffold on ChE inhibition were explored by Hasan et al., (Figure 5) [39]. Depending on the substitution patterns on the two aromatic rings, the inhibitory potential was varied. The AChE inhibition was in the range of 28.2–134.5 μM, while BChE inhibition was in the range of 16.0–23.1 μM. From the results, it is evident that the -OH group at ring A (ortho to the carbonyl attachment) has a promising role in AChE inhibition. Unsubstituted analogs exerted weaker activity than the ortho-substituted-OH functionalities at ring A. Further, the presence of -Cl at ring A resulted in reduced activity. Moreover, depending on the number of -OCH_3_ functionalities, the AChE inhibition was varied, wherein di-substituted analogs exerted higher activities than mono substitution. Amongst the numerous substitutions on B rings, ortho substitution was found to be the most promising position for BChE inhibition. The meta and para substitutions on the B ring resulted in decreased activities. Further, the introduction of methoxy in the meta and para positions also exerted beneficial effects on the BChE inhibitory potential [39].

**Figure 6:**
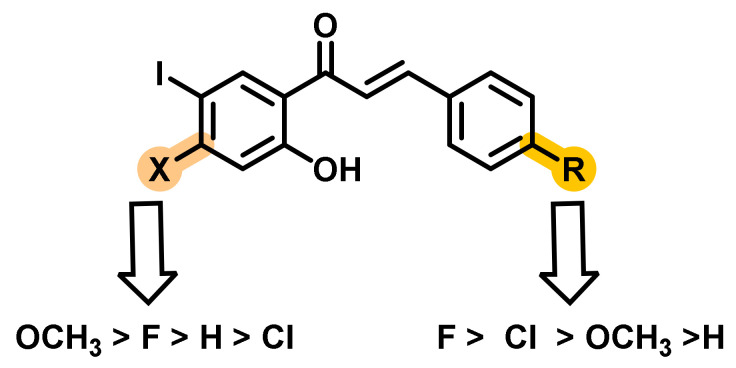
Chalcone derivatives with simple substitutions by Mphahlele et al. [40].

Mphahlele et al., synthesized and evaluated the ChEs inhibitory potential of a series of 4-substituted 2-hydroxy-5-iodochalcones (Figure 6) [40]. Except for two analogs, all the remaining analogs exerted moderate to good inhibitory potential (9.38–11.56 μM) for AChE inhibition. Donepezil was used as a positive control, which exerted an activity of 4.77 μM. On the contrary, some analogs exerted higher BChE inhibition (4.17 to 5.73 μM) than the standard drug donepezil (6.04 μM). A combination of the lipophilic 4-methoxyphenyl group (B ring) and fluorine, chlorine, or methoxy substituent at the 4-position of ring A seems to be more favorable for BChE inhibitory activity. However, a combination of the 4-bromo/methoxy substituent on ring A and a 4-fluorophenyl group at the β-carbon resulted in the potent BChE inhibitor (IC_50_ = 5.73 μM). It is also interesting to note that the anti-cholinesterase activity is proportional to the decreasing propensity of the 2-(4-substituted phenyl) ring, wherein the delocalization of π-electron into the α,β-unsaturated framework significantly varied. Further, the most active chalcone derivatives against the BChE tend to be moderately inhibiting against AChE [40].

**Figure 7:**
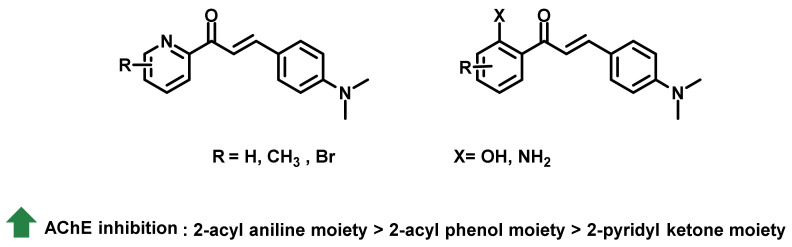
Chalcone derivatives with simple substitutions by Fosso et al. [41].

Fosso et al., synthesized different classes of chalcones and biochemically evaluated their in vitro AChE inhibitory potential. These main classes were mainly designed by considering the metal chelating ability of numerous chemical scaffolds, and include 2-pyridyl ketone, 2-acyl phenol, and 2-acyl aniline moieties (Figure 7) [41]. All of the tested chalcones displayed micromolar IC_50_ values: 2-pyridyl ketone-containing analogs exerted activities of 9.55 μM to > 200 μM, followed by 2-acyl phenol-containing analogs (1.61 μM to > 200 μM). The most potent activities were exhibited by 2-acyl aniline moieties (IC_50_ = 0.503 μM to 4.0 μM). In the SAR perspective, for the pyridyl ring, methyl substitution did not exert any improvement in the inhibitory activity. Meanwhile, the attachment of -Br at the 2nd and 3rd position of pyridyl ring resulted in enhanced inhibitory activity. The replacement of 2-pyridyl ketone with 2-acyl phenol resulted in a slight enhancement of AChE inhibition. However, further substitution did not impart any inhibitory effects. Further substitution of 2-acyl aniline moieties resulted in potent activities. These analogs exerted lesser potential towards BChE inhibition. It is noteworthy that the presence of metal ions resulted in decreased activities by these analogs against ChEs [41].

**Figure 8:**
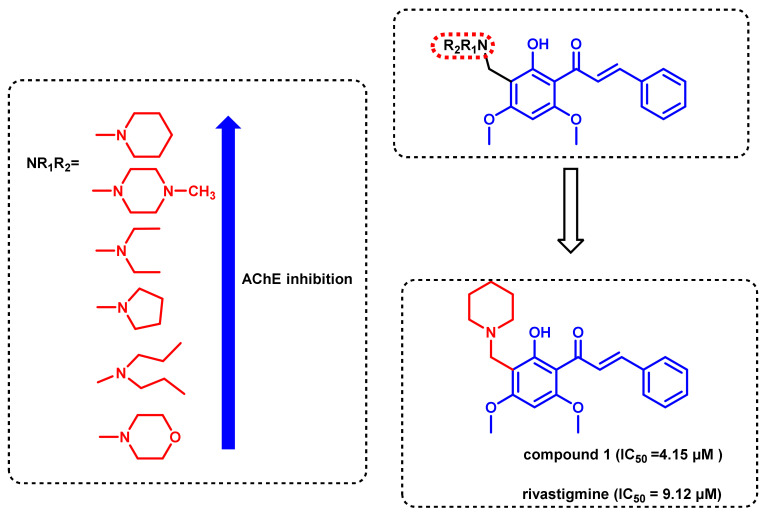
Chalcones inspired from Flavokawain Mannich bases.

Flavokawain B, a natural chalcone derivative isolated from the roots of kava, showed promising pharmacological activities, but its studies were limited due to their low yields. Inspired by the promising role of Flavokawain B, Liu et al., reported the synthesis and AChE inhibitory activities of a series of Flavokawain Mannich bases (Figure 8) [42]. The various substitutions were made on the amino methylene group, ranging from dimethylamine to morpholine, which played a crucial role in determining the inhibitory potential of each derivative. Compound 1 with piperidine substitution on the amino methylene group showed potent inhibition of AChE with an IC_50_ value of 4.15 μM, which was a 2-fold increase in activity when compared to the standard rivastigmine (IC_50_ = 9.12 μM). Substitution with a hydrophobic group such as morpholine showed a drastic decrease in activity. The logP value of the active analogs was less than 2 ± 0.7 (1.58 to 1.77), suggesting the lipophilicity to pass the BBB. The docking studies of compound 1 with AChE (PDB ID:1EVE) supported the biological results, as it showed important interactions including π-π interactions of chalcone with Trp 29 and Tyr 334 of the PAS and cation-π interactions of piperidine nitrogen with Trp 84 and Phe 330.

Based on the potential role of amino functionalities in ChE inhibition, numerous groups have attempted the exploration of these substituents as a promising inhibitor. Numerous approaches have been made, such as the incorporation of other substituents along with amino substituents, the modification of open amino to rigid amine derivatives, and interchanging amine to sulphonamide. These kinds of structural modifications resulted in varied pharmacological potential.

**Figure 9:**
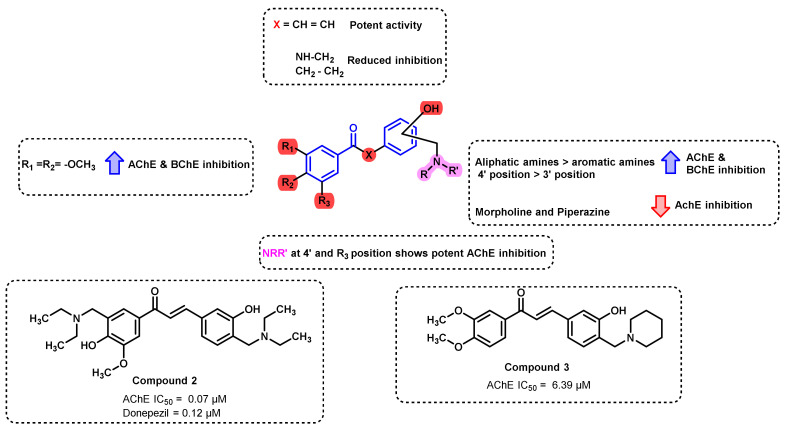
Mannich bases-derived chalcone reported by Zhang et al.

The effect of amino functionality on the ChE inhibition was established by a multidrug design strategy, wherein a series of chalcone Mannich bases was reported by Zhang et al., (Figure 9) [43]. The evaluation of AChE and BChE inhibitory potential revealed the important structural activity relationships (SAR) of chalcone Mannich bases and suggested that aliphatic amines showed more potent activity than aromatic amines. Cyclic amine derivatives (such as morpholine and piperazine) result in a decreased inhibition of both enzymes, whereas methoxy substitution on chalcones (especially in ring A) had a significant role in the activity. Para-position-substituted amines showed a more potent inhibition profile than meta-position substitution. Further, the conversion of chalcone (comprising unsaturated functionality) to simple amides resulted in the reduction of ChEs-inhibitory potential. Derivatives containing two amino groups (bis Mannich derivatives) on chalcones showed potent AChE inhibition (compound 2; IC_50_ = 0.07 µM), whereas compound 3 (IC_50_ = 6.39 µM) with alkylamine substitution showed potent BChE inhibition. Donepezil was used as a positive control that exerted activity of 0.12 and 20.7 µM, respectively, towards AChE and BChE inhibition. PAMPA-BBB assay of compound 2 indicated its high potential to cross the BBB (permeability, Pe-13.5 × 10^−6^ cm/s) [43].

**Figure 10:**
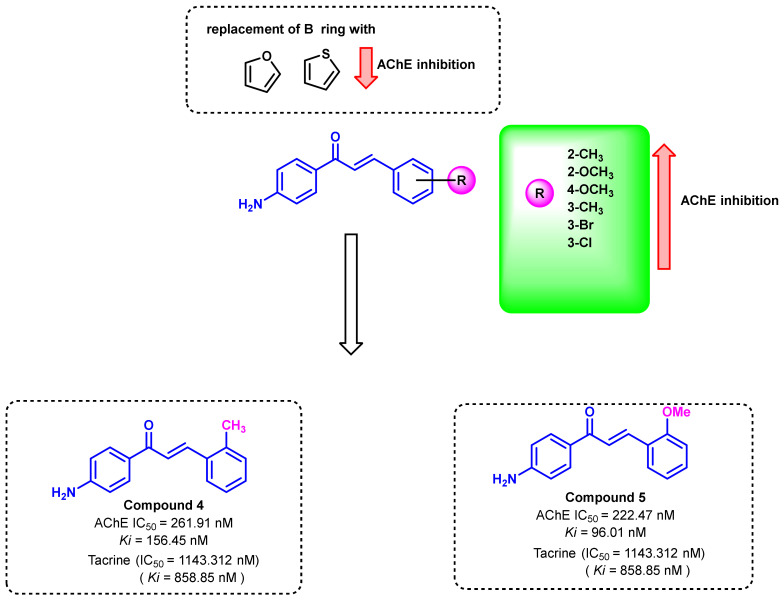
4-amino chalcone derivatives reported by Gurdere et al.

A series of 4-amino chalcones were synthesized by Gurdere et al., by replacing the B ring with various substituted phenyl and heterocyclic rings (Figure 10) [44]. All of the synthesized compounds showed higher potency for AChE inhibition (222.47–1048.41 nM) than the standard tacrine (IC_50_ = 1143.312 nM), but none of the compounds showed good activity against BChE. The phenyl ring substituted with EDG demonstrated a higher potency (compounds 4 and 5). The replacement of the phenyl B ring with heterocyclic rings like furan and thiophene decreased the ChEs inhibitory activities to a larger extent [44].

**Figure 11:**
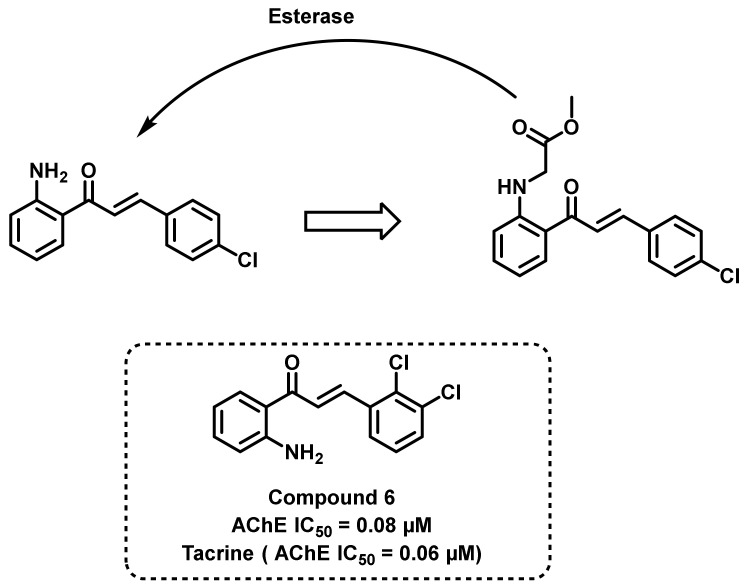
2′-amino chalcones derivatives reported by Sakata et al.

Sakata et al., synthesized a series of 2′-aminochalcones and evaluated their AChE inhibitory potential (Figure 11) [45]. Compound 6 exerted the most potential activity (IC_50_ = 0.08 µM) compared to tacrine, which was used as a positive control (IC_50_ = 0.06 µM). However, these analogs exerted poor BChE inhibition. Molecular docking studies (PDB ID = 1EVE) were also in agreement with the in vitro results. The synthesized amino chalcones demonstrated poor aqueous solubility. Substitution of the amino functionalities with a methyl ester (by treating with methyl glycinate) resulted in the enhancement of AChE inhibitory potential (0.08–8.34 µM). The molecular descriptor-based results revealed that the majority of these compounds exerted moderate BBB permeability (Log BB–0 to 0.5) [45].

### 2.2. Chalcone Derivatives with Amine Substituent Modification

**Figure 12:**
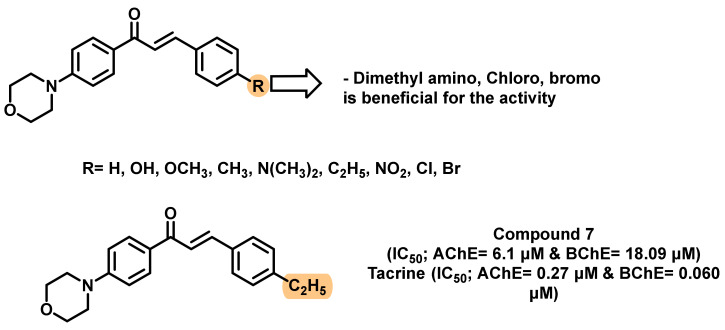
Structural refinement involved the modification of simple amino to cyclic morpholine derivatives.

The modification of a simple amino to the denser cyclic functionalities has been reported by various groups. The ChEs inhibitory potential of various morpholine-based chalcone analogs was reported by Sasidharan et al., (Figure 12) [46]. Among the screened analogs, compound 7 exerted the maximum inhibitory potential (IC_50_; AChE = 6.1 μM, BChE = 18.09 μM). Tacrine was used as a positive control (IC_50_; AChE = 0.27 μM, BChE = 0.060 μM). The presence of dimethylamino, chloro, and bromo substituents at the para position on the chalcone B ring conferred moderate AChE inhibition. Further, these analogs exhibited weaker BChE inhibition (>40 μM). PAMPA-BBB assay suggested their higher membrane permeability to brain (Pe-14.44 to 16.34 × 10^−6^ cm/s) [46].

**Figure 13:**
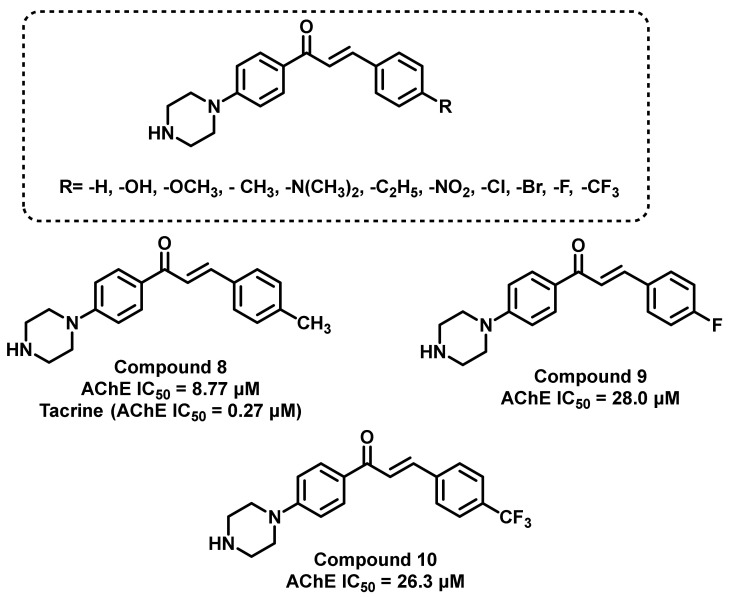
Structural refinement involved the modification of simple amino to cyclic piperazine derivatives.

In another study, a series of piperazine-substituted chalcone was synthesized and their AChE inhibitory potential evaluated by Mathew et al., (Figure 13) [47]. Compound 8 exhibited the maximum potential (IC_50_ = 8.77 μM), followed by compound 9 and compound 10 (IC_50_ = 28.0 and 26.3 μM, respectively), and tacrine was used as a standard compound (IC_50_ = 0.27 μM). Further molecular docking analysis (PDB ID: 4EY7) demonstrated a positive correlation between the docking score with the inhibitory activity. The ADME prediction of these analogs suggested higher BBB permeation properties [47].

**Figure 14:**
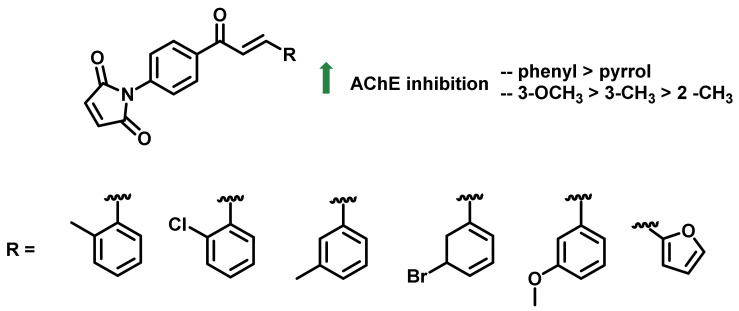
Chemical structures of chalcone imide derivatives.

Kocyigit et al., synthesized a series of chalcone imide derivatives and evaluated their AChE inhibitory potential (Figure 14). The newly synthesized series comprised chalcones with pyrrole scaffolds. The reaction of 4′-aminochalcone derivatives with maleic anhydride in the presence of triethylamine resulted in the formation of pyrrole-appended chalcones. The screened analogs exerted good AChE inhibitory activities (K_i_ values were in the ranges of 70.470–229.42 nM) and tacrine was used as a positive control (K_i_ = 446.56 nM) [48].

**Figure 15:**
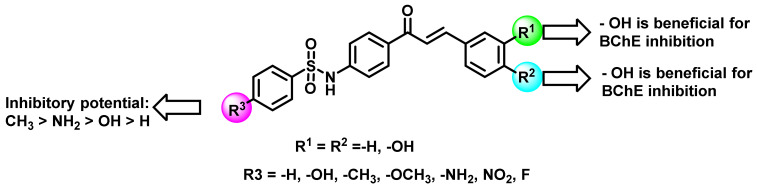
Chemical structures of chalcone sulphonamide derivatives.

Kang et al., synthesized a series of sulfonamide-containing chalcones and evaluated their ChE inhibition properties (Figure 15) [49]. The majority of the synthesized analogs exerted more potential activity towards BChE than AChE inhibition (K_i_ value = 9.8 to 72.2 and 40.4 to 80.3 µM, respectively, for BChE and AChE inhibition). Further analysis showed all ChE inhibitors were reversible and exhibited a mixed inhibition kinetics nature.

### 2.3. Chalcone Derivatives with Hydroxyl Substituent Modification

From the previous parts, it is evident that the incorporation of hydroxyl functionalities resulted in an enhanced ChE inhibitory activity of chalcones. Derived from these observations, numerous efforts have occurred for examining the effects of modification (hydroxyl group into other functional groups including ethers, esters, and amides) in ChEs inhibitory potential. Amongst these structural modifications, ether-based chalcones showed a better inhibitory profile against ChE.

**Figure 16:**
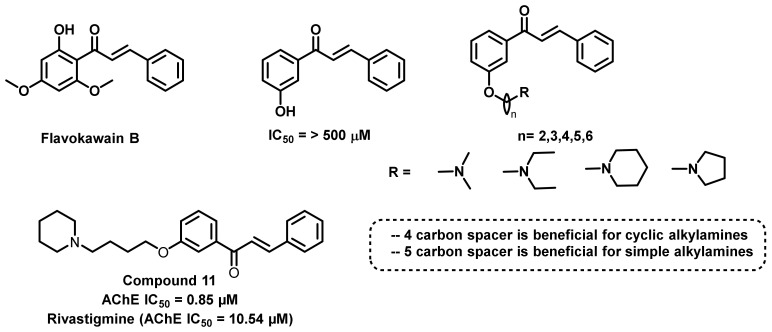
Ether-substituted chalcone derivatives reported by Liu et al.

A new series of derivatives was synthesized and their ChEs inhibitory potential evaluated by Liu et al., (Figure 16) [50]. The described work mainly evaluated the effects of the replacement of hydroxyl functionalities to ether (connected to tertiary amine groups) in the ChE inhibition. Compound 11 displayed the strongest AChE inhibitory activity (IC_50_ = 0.85 µM) and highest selectivity (ratio: 35:79), and rivastigmine was used as a positive control (IC_50_ = 10.54 µM). The alteration in the amino alkyl side chain and chalcone scaffold resulted in a drastic difference in the activity as well as the selectivity. For the simple alkyl chains (dimethylamine and diethylamine), the five carbon spacer resulted in the potential activity. However, for the cyclic chains (pyrrolidine and piperidine), the four carbon spacer revealed the maximum AChE inhibition. The three carbon spacer showed the poorest inhibition towards the screened enzymes. The kinetic enzyme inhibition of compound 11 revealed a mixed type of inhibition. Molecular docking was also in accordance with the obtained in vitro activities (AChE PDB ID = 1EVE). Further, the synthesized compounds demonstrated a logP value of 1.54 to 1.98, suggesting its higher brain permeation properties [50].

**Figure 17:**
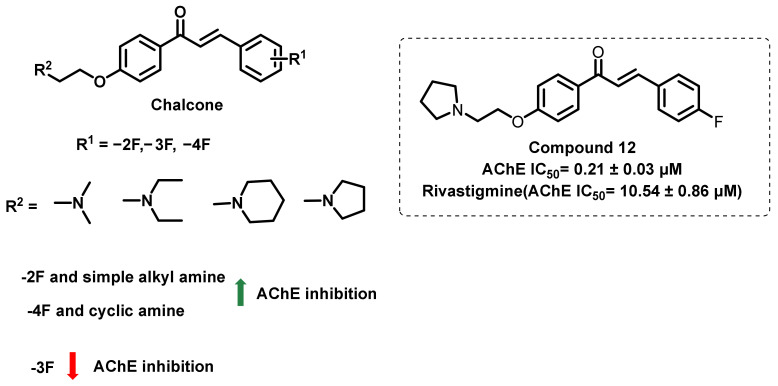
Fluoro-chalcone-substituted amino alkyl derivatives.

In another study, Liu et al., synthesized a new series of fluoro-chalcone-substituted amino alkyl derivatives (Figure 17) [51]. The alteration of the fluorine atom position and amino alkyl groups markedly influenced the activity and the selectivity of the chalcone derivatives in AChE and BChE inhibition. Compound 12 possessed the most potent inhibitory against AChE (IC_50_ = 0.21 ± 0.03 μM), and the highest selectivity for AChE over BChE (IC_50_ (BChE)/IC_50_ (AChE) = 65.0). Amongst the screened analogs, the 3-fluoro-substituted analogs exerted weaker activity than the 2- and 4-fluoro-substituted analogs. In the simple alkyl chain analogs, the 2-fluoro-substituted analogs exerted higher AChE inhibition than the 4-fluoro substitutions, while for cyclic amine functionalities (piperidine- and pyrrolidine-containing amino acids), the 4-fluoro-substituted chalcones exerted higher activity than the 2-fluoro-substituted derivatives. Moreover, 4-fluoro substitution revealed a higher selectivity towards AChE inhibition than BChE inhibition. The enzyme kinetic analysis revealed a mixed type of inhibition by these analogs. Moreover, these compounds showed a logP of 1.65 to 1.80, suggesting the potential BBB permeation [51].

**Figure 18:**
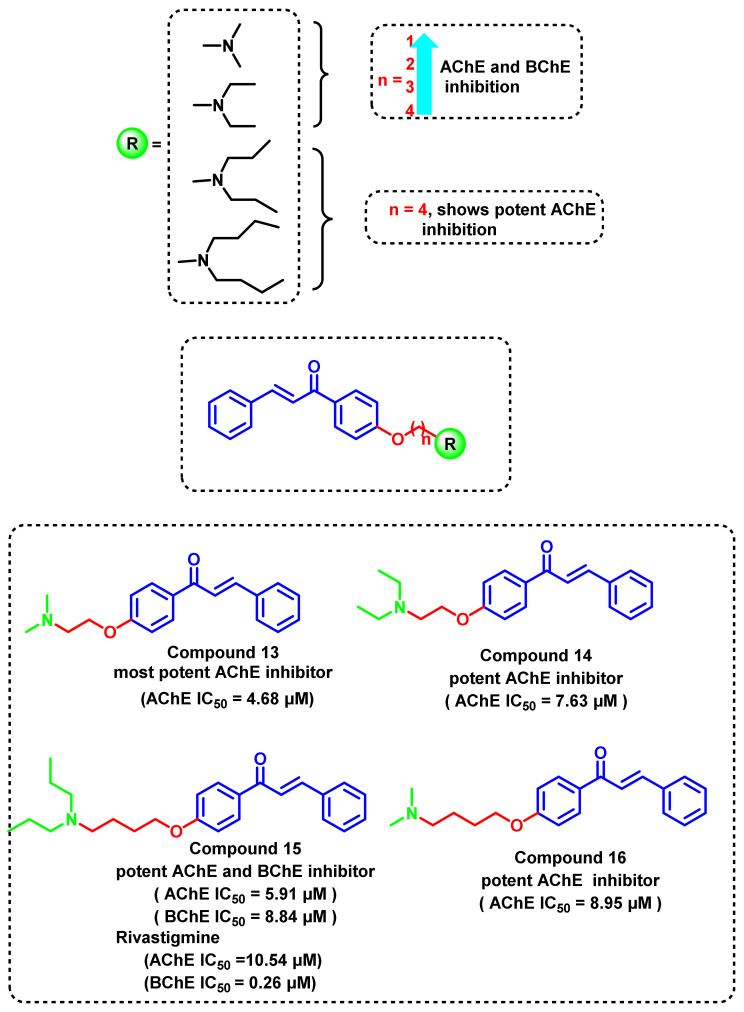
Open-chain amino alkyl chalcone derivative.

A series of chalcone derivatives was reported by Liu et al., (2014) with different amino side chains on the para position (Figure 18) [52]. The amino groups were linked to the chalcone moiety via a carbon spacer of varying lengths (n = 2–6). The influence of chain length on the ChEs inhibitory activities (4.68–8.95 μM) was evident from the biological studies. In the dimethylamine and diethylamine series of compounds, the activities against AChE and BChE decreased with an increase in linker length. In general compounds with an even number of carbon atoms as a spacer showed potent AChE inhibition. In the series of dipropylamine and dibutylamine, the compounds with a four-carbon linker showed potent AChE inhibition. The possibility of these analogs has been confirmed from its logP value (1.49 to 2.19) [52].

**Figure 19:**
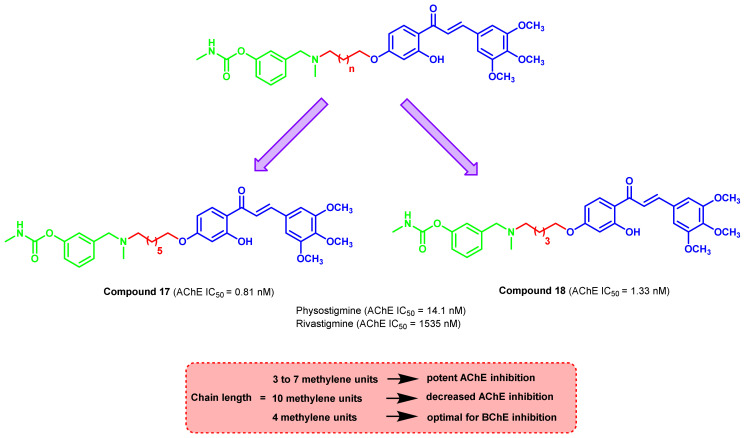
Chalcone–carbamate hybrid analogs.

Carbamates have always been a molecule of interest due to their chemical stability and increased permeability across the cell membrane. A series of chalcone carbamates was designed by varying the linker length (Figure 19) [53]. The in vitro inhibition studies on AChE suggested the varying effects of linker length between the carbamate and chalcone scaffold. The incorporation of 10 methylene units resulted in a decremental activity, while a 3 to 7 spacer demonstrated beneficial ChEs inhibitory potential. All of the screened compounds showed nanomolar potency (0.52 to 51.8 nM), which was much higher than those of standards, rivastigmine (AChE IC_50_ = 1535 nM), and physostigmine (AChE IC_50_ = 14.1 nM). Compound 17 showed the highest potency with an IC_50_ value of 0.81 nM for AChE inhibition. Compound 18 showed significant activities towards both enzymes (AChE IC_50_= 1.33 nM, BChE IC_50_ = 30.2 nM).

**Figure 20:**
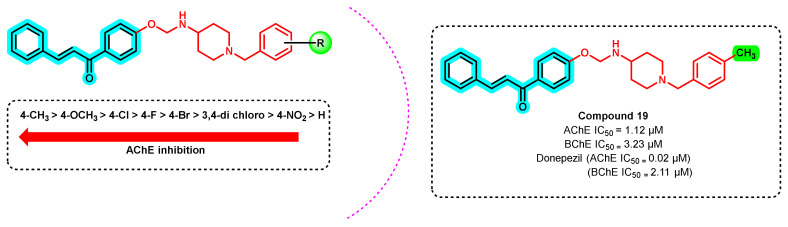
Chalcone benzyl piperidine derivatives.

A series of chalcone–benzyl piperidine derivatives was synthesized using an alkyl amine linker and the effect of various substituents on the ChE inhibitory activity was evaluated (Figure 20) [54]. The unsubstituted benzyl counterpart of the benzyl piperidine resulted in poor ChEs inhibition, highlighting the importance of substitution on the latter. Para-substituted derivatives demonstrated a potent inhibition, wherein compound 19 showed potent inhibition against both enzymes with an SI value of 2.88 towards AChE. Electron-donating substituents such as methyl and methoxy groups showed better prominent inhibition than the electron-withdrawing halogens (-Cl, -F) [54].

**Figure 21:**
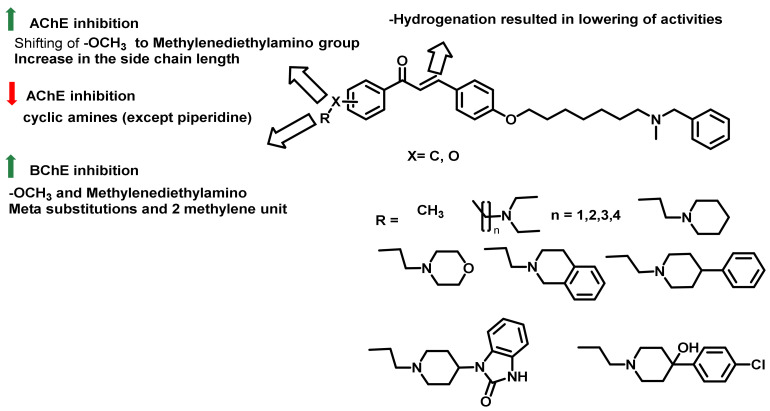
Chalcone derivatives reported by Rampa et al.

Naturally inspired chalcone-based derivatives were designed and synthesized by Rampa et al., who evaluated the inhibitory activities against ChEs (Figure 21) [55]. A ring of chalcone was decorated with a methoxy or an additional tertiary amino functionality (at the para or meta positions). Further, the role of α,β-double bond was evaluated by hydrogenation, resulting in the formation of more flexible analogs. The introduction of the methoxy group in the chalcone resulted in decreased AChE activity. The replacement of methoxy with methylenediethylamino functionalities demonstrated an increase in inhibitory activities (0.70 to 511 µM for AChE). Further increments in the side chain length (2 to 4 carbon) allowed enhanced AChE activity. The replacement of simple amines with bulkier amines resulted in the reduction of ChEs activities. Further, the hydrogenation of α,β-double bond demonstrated a weakening of the AChE inhibitory activity. Contrary results were demonstrated in BChE inhibition, wherein methoxy functionalities led to the increase in potencies. Moreover, meta-substituted alkoxy amino functionalities resulted in the potential analogs. Hydrogenated analogs resulted in a three- to four-fold reduction in BChE inhibition [55].

**Figure 22:**
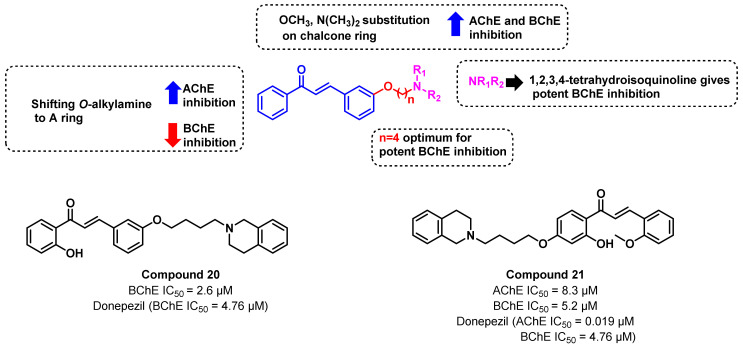
O-alkylamine-substituted chalcone derivatives reported by Sang et al.

In another study by Sang et al., a multi-design strategy was adopted, wherein *O*-alkylamine functionalities were incorporated into the chalcones (Figure 22) [56]. Compared to the simple chalcones, *O*-alkylamine-substituted chalcone showed a moderate to good inhibition against AChE and BChE, that further concealed the importance of a multi-design strategy. The length of the alkyl chain, as well as the terminal amine group, significantly influenced the BChE-inhibitory activity. Substitution of 1,2,3,4-tetrahydroisoquinoline as the amine functionality of alkylamine led to potent BChE inhibition (compound structure and number). An optimal chain length of four methylene units resulted in potent BChE inhibition, whereas shifting the *O*-alkylamine to the A ring significantly decreased the BChE inhibition, but increased the AChE-inhibitory activity. The presence of EDG such as -OCH_3_ and N(CH_3_)_2_ was important for AChE/BChE inhibition, whereas EWG decreased the ChE-inhibitory activity. The permeability, Pe, of the synthesized compounds lies in the range of 10.02 to 16.79 × 10^−6^ cm/s, suggesting its potential BBB permeation [56].

**Figure 23:**
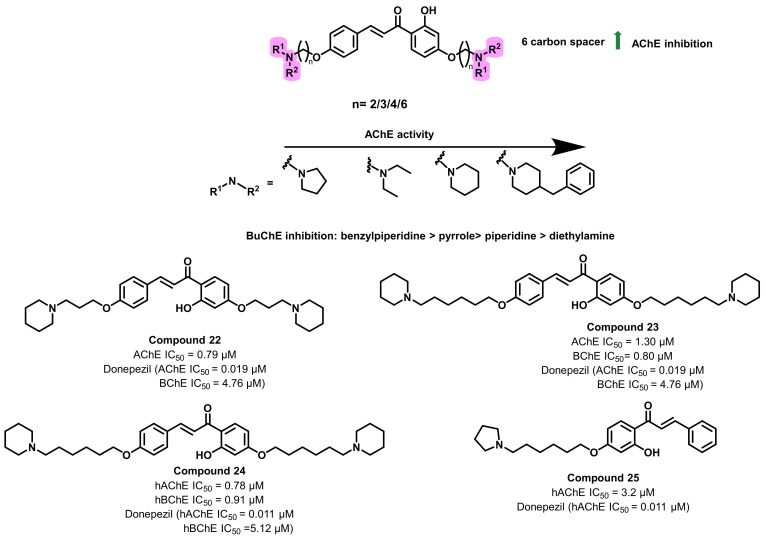
Chalcone-*O*-alkylamine derivatives reported by Bai et al.

The design and synthesis of a series of chalcone-*O*-alkylamine derivatives were reported by Bai et al., (Figure 23) [57]. Synthesis mainly involved the alkylation of the 4′-OH of 2′,4′-dihydroxyacetophenone using various dibromo alkanes of varying chain length (n = 2 to 6). Further treatment of these halides with secondary amines resulted in alkylamine-substituted acetophenone derivatives. Alkyl amine-substituted aldehydes were also prepared in the same manner. The targeted derivatives were prepared via treating the alkyl amine-substituted aldehydes with acetophenone derivatives under basic conditions. The in-vitro screening of the synthesized analogs revealed moderate to good inhibitory activities against AChE and BChE (0.79 to 23.90 µM and 0.80 to 50.2 µM, respectively). The length of the methylene chain and the structure of terminal groups NR_1_R_2_ of the side chain significantly affected the AChE- and BChE-inhibitory activities. A direct correlation between the methylene chain length and AChE-inhibitory activity was exhibited, wherein the optimum length was found to be six. In most conditions, when the length of the methylene chain remained the same, the potency to inhibit AChE was in the order: pyrrole > diethylamine > piperidine > benzylpiperidine. Compound 22 showed the best AChE inhibitory activity with an IC_50_ value of 0.79 µM. A similar trend was observed in the case of BChE inhibition, in the order of benzylpiperidine, piperidine, and pyrrolidine. Compound 23 showed the best BChE-inhibitory activity with an IC_50_ value of 0.80 µM. The titled compounds lacked the selectivity towards AChE and BChE. Thus, further structural modifications were performed, wherein the symmetrical *O*-alkylamine fragment was removed. This kind of structural modification resulted in decreased ChEs inhibitory potential. However, the selectivity towards the ChEs increased (2.9 to 5.6). The potential of the most active analogs was further investigated in human AChE (hAChE) and hBChE by using donepezil as a standard drug. Compound 24 displayed good hAChE- and hBChE-inhibitory activities, with IC_50_ values of 0.78 µM and 0.91 µM, respectively, and compound 25 showed good hAChE-inhibitory activity with an IC_50_ value of 3.2 µM, implying that AChE-inhibitory activity by human AChE was better than that by *Electrophorus electricus* AChE (eeAChE). Molecular docking studies with AChE (PDB code: 1EVE) and BChE (PDB code: 4TPK) highlighted that these compounds occupied the entire catalytic active sites. Most active analogs demonstrated a Pe value of 9.68 × 10^−6^ cm/s, suggesting its potential BBB permeation [57].

**Figure 24:**
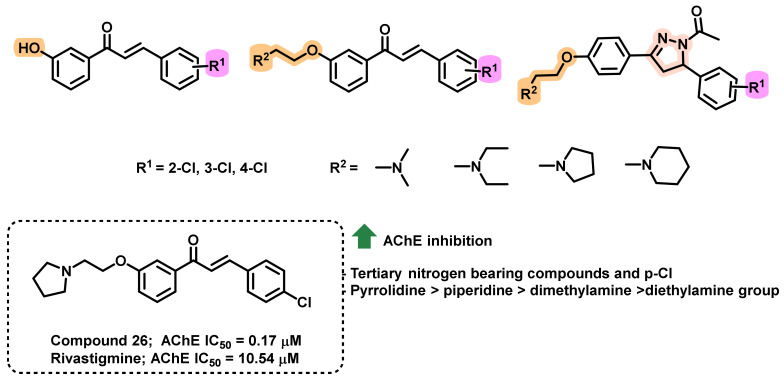
Chlorochalcones with tertiary amine side chain.

Inspired from the chalcones bearing tertiary nitrogen atoms and numerous halogen atoms, Gao et al., synthesized a series of chlorochalcones with a tertiary amine side chain (Figure 24) [58]. In order to evaluate the role of α,β-unsaturated ketone groups in ChE inhibition, pyrazoline-containing analogs were also synthesized. All of the amino alkyl-substituted chlorochalcones exerted better inhibitory activity against the AChE than the unsubstituted counterparts (0.17–3.76 μM). Further, variation in the amino alkyl side chain markedly influenced the AChE inhibition (pyrrolidine > piperidine > dimethylamine > diethylamine group). Moreover, the position of the chlorine atom also revealed a variation in the ChE inhibition. Para-substituted chlorochalcone derivatives had the highest selectivity in inhibiting AChE over BChE. Furthermore, for the alkyl amino-terminal groups (dimethylamine or diethylamine), the order of inhibitory potency against AChE was: para > meta > ortho, while for cyclic amino-terminal groups (piperidine or pyrrolidine), the activity was in the order of para > ortho > meta. Compound 26 exerted the maximum activity (IC_50_ = 0.17 μM) compared to rivastigmine (IC_50_ = 10.54 μM). Further, this analog demonstrated a mixed type of inhibition with AChE. These analogs exerted weaker activity against BChE (IC_50_ in the ranges of 27.62 to > 500 μM). Cyclization of α,β-unsaturated ketones resulted in the formation of pyrazoline derivatives, that exerted weaker inhibitory activity towards ChEs. These results suggested the importance of α,β-unsaturated ketones in the ChEs inhibition. Molecular docking studies (PDB ID: 1EVE) were also in accordance with their in vitro inhibitory activities. The logP values of the synthesized analogs ranged from 1.61 to 1.83, indicating the lipophilicity of these analogs for crossing the BBB [58].

## 3. Chalcone-Based Hybrid with USFDA-Approved Drugs

The multitargeted approach has become a prominent scientific approach to treat various neurodegenerative diseases [59]. Chalcones with effective AChE inhibiting potential have been combined with various USFDA-approved drugs for better efficacy and less toxicity.

**Figure 25:**
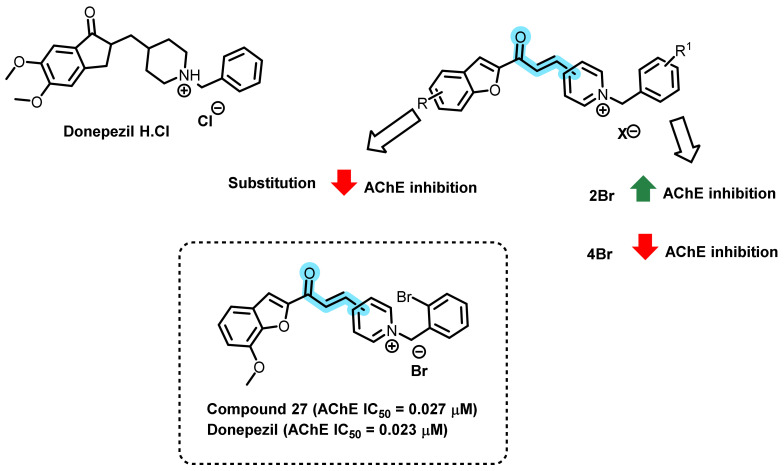
Benzofuran-based chalconoid-containing N-benzylpyridinium motif.

Mostofi et al., designed and synthesized a series of benzofuran-based chalconoid-containing N-benzylpyridinium motif and evaluated AChE inhibitory potential (Figure 25) [60]. Most of the analogs had significant anti-AChE activity at micromolar or sub-micromolar levels. Amongst the 3-pyridinium salts and 4-pyridinium salts, a variable activity was observed. Bromine substitution at the second position of benzyl moiety resulted in the enhancement of AChE inhibition. On the contrary, 4-Br substituent resulted in the decrement in the activity. Substitution at the 5- or 7-position of the benzofuran ring was generally not tolerated. However, compound 27 was the preferred 2-bromobenzyl substituent, which led to the tolerance of the 7-methoxy substituent on the benzofuran ring, providing the most potent compound in the series (IC_50_ = 0.027 µM) [60].

**Figure 26:**
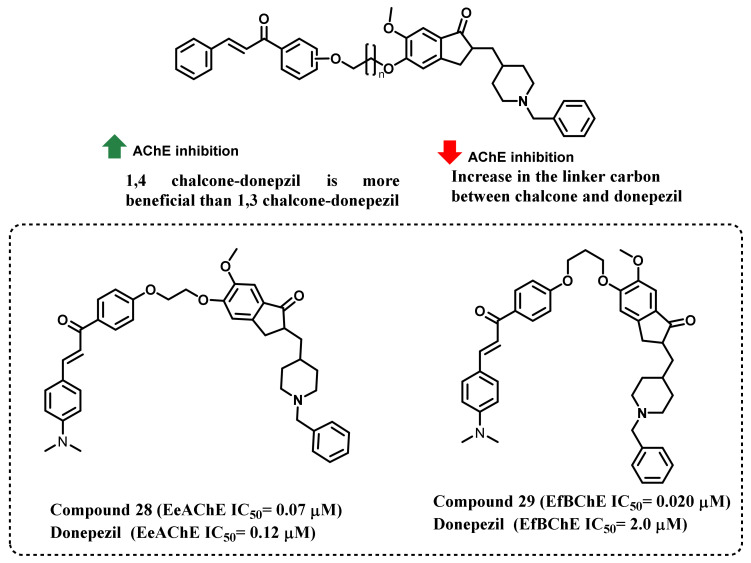
Chemical structures of donepezil–chalcone hybrid derivatives.

Chandrika et al., synthesized a new series of donepezil–chalcone hybrids and evaluated their ChEs inhibitory activities against *Electrophorus electricus* (EeAChE for AChE inhibition) and *Equus ferus* (EfBChE for BChE activity) (Figure 26) [61]. Numerous chalcones such as 1,3- and 1,4-substitutes were incorporated into the donepezil and those were separated with numerous carbon chain linkers (2, 4, 6, 8, 10, and 12 carbons). 1,4-Chalcone–donepezil hybrids were more effective at inhibiting EeAChE. In the case of EfBChE inhibition, in shorter carbon linkers (2 and 4 carbons), 1,3-chalcone-based hybrid exerted higher inhibitory potential, while 1,4-chalcone-based hybrid exerted higher inhibitory potential in the longer carbon chain spacers (6 to 12). An increase in the linker length from 2 to 12 carbons resulted in a gradual increase of IC_50_ values. Donepezil exerted activity of 0.12 and 2.0 µM activity, respectively, against EeAChE and EfBChE. Compound 28 exerted the maximum EeAChE activity of 0.07 µM (IC_50_ value), while compound 29 exerted the maximum EfBChE inhibition, with an IC_50_ value of 0.020 µM [61].

**Figure 27:**
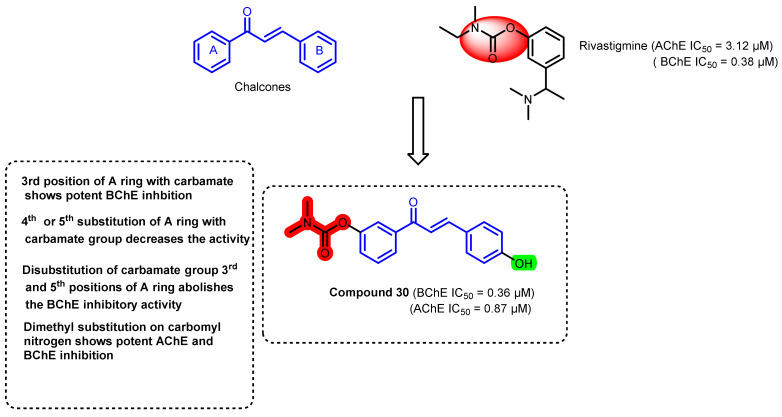
Chemical structures of rivastigmine–chalcone hybrid derivatives.

Another synthetic approach involving the design of the chalcone–rivastigmine hybrid was carried out by Wang et al., (Figure 27) [62]. Most of the compounds were active against BChE, while some of the compounds showed a potential AChE inhibitory profile. Substitution of the active carbamate group at the 3rd position of the A ring of chalcone showed potent BChE inhibition (compound 30, IC_50_ = 0.36 μM), while the 4th and 5th substitutions of the carbamate group decreased the activity. The disubstitution of the carbamate group at the 3rd and 5th positions of the A ring abolished the BChE inhibitory activity. Alkyl substitution on carbamoyl nitrogen also affected the inhibitory profile and dimethyl substitution showed potent BChE and AChE inhibition. Except a few analogs, these molecules revealed poor BBB-crossing properties [62].

**Figure 28:**
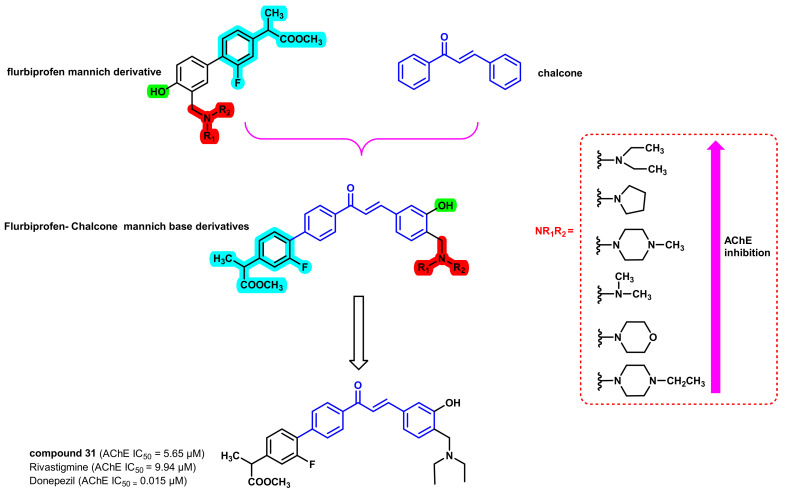
Chemical structures of flurbiprofen Mannich derivative–chalcone hybrid derivatives.

The combination of the flurbiprofen Mannich derivative and chalcone moiety resulted in hybrid chalcone analogs (Figure 28) and the derivatives were screened for their multi-drug activities, including AChE inhibition relating to β-amyloid (Aβ) aggregation [63]. Various small molecular-weight amines were substituted on the 4th position of the chalcone B ring. All of the synthesized analogs showed potent inhibition of AChE without BChE inhibition (7.15 to 35.65 µM for EeAchE). Compound 31 with a diethylamine side chain showed the most potent AChE inhibition with a Pe value of 6.21 × 10^−6^ cm/s [63].

**Figure 29:**
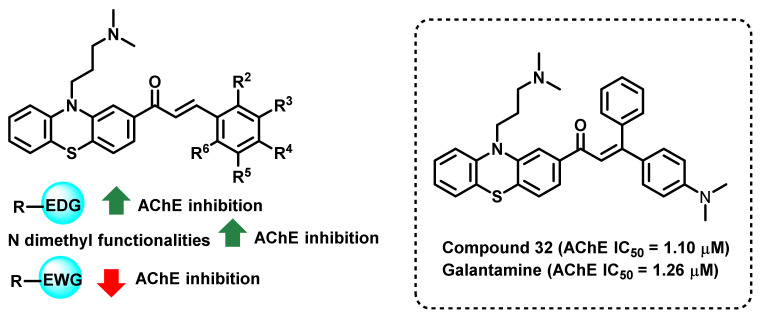
N-substituted-4-phenothiazine chalcone derivatives.

Tran et al., synthesized a series of *N*-substituted-4-phenothiazine-chalcones and evaluated their AChE inhibitory potential (Figure 29) [64]. The synthesized analogs demonstrated an AChE inhibitory potential in the range of 1.10 to 186.21 µM. *N*-Dimethyl-substituted analogs (compound 32) exerted higher activity (IC_50_ = 1.10 µM) than the standard drug (galantamine, IC_50_ = 1.26 µM). It is also noteworthy that the authors developed a two-dimensional quantitative structure–activity relationship (2D-QSAR) model for AChE inhibition (squared correlation coefficient, R^2^ value = 0.70 and cross validated squared correlation coefficient, Q^2^ value = 0.57). Based on the developed SAR and molecular docking (PDB ID: 1DX6) approach, a correlation was drawn for the potential for AChE inhibition [64].

**Figure 30:**
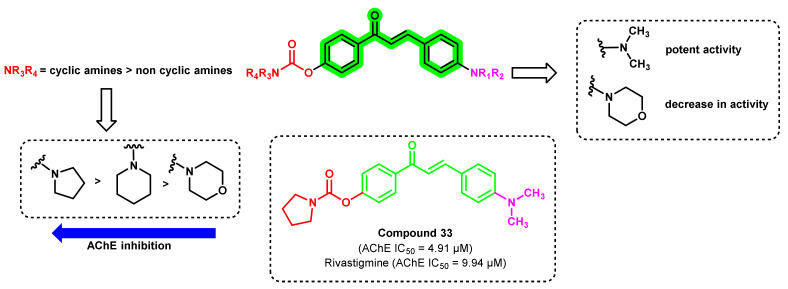
Chemical structures of 4-amino chalcone-rivastigmine derivatives.

A hybrid of 4-amino chalcone and rivastigmine was synthesized and evaluated for its anticholinesterase, antioxidant, and metal-chelating properties (Figure 30) [65]. *N, N*-disubstituted carbamate pharmacophore of rivastigmine was combined with an amino chalcone. The introduction of different amino groups on carbamoyl moiety with dimethyl amino group on the 4-position of chalcone scaffold (ring B) showed varying results. Cyclic amines showed better inhibition of AChE than noncyclic amines. Among the cyclic amines, the pyrrolidine ring showed potent inhibition (compound 33, IC_50_ = 4.91 μM). Changing the dimethylamino group on the 4-position with the morpholine ring greatly reduced the activity by several folds. The dual substitution of the carbamate group on 2′ and 4′ positions also reduced the activity. An increment in the overall size, as well as the hydrophilicity, might be the probable reason for the reduction in ChEs inhibitory potential. Further, compound 33 exhibited a Pe value of 7.03 × 10^−6^ cm/s, suggesting its higher BBB permeation properties [65].

## 4. Miscellaneous

### 4.1. Natural Chalcones

**Figure 31:**
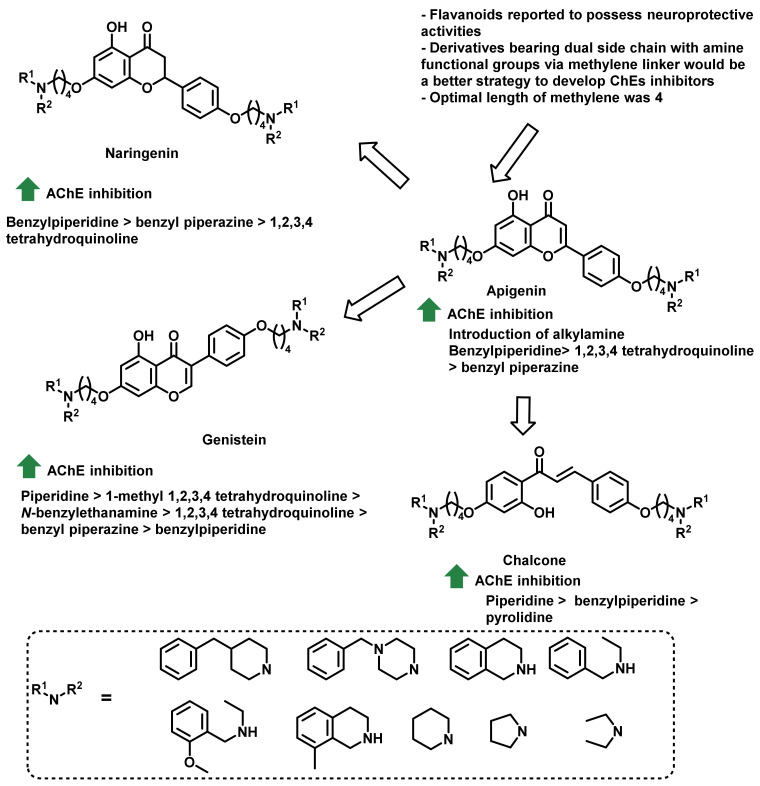
Flavonoid-inspired chalcone derivatives.

Inspired from the wide range of pharmacological benefits of flavonoids, Sang et al., synthesized a series of hybrid analogs (Figure 31) [66]. Apigenin, naringenin, and genistein were utilized as the starting template. Other pharmacophoric features such as dual side chain with amine functional groups via methylene linker have also been included in the designed analogs. All of the synthesized analogs exerted moderate to good inhibitory activities (IC_50_ ranges from 0.52 to 26.7 µM against eeAChE and eqBChE from equine serum). Parent flavonoids did not exert any kind of ChE inhibition. In the PAMPA-BBB assay, the most active analog showed a greater permeability (Pe: 15.72 × 10^−6^ cm/s) [66].

**Figure 32:**
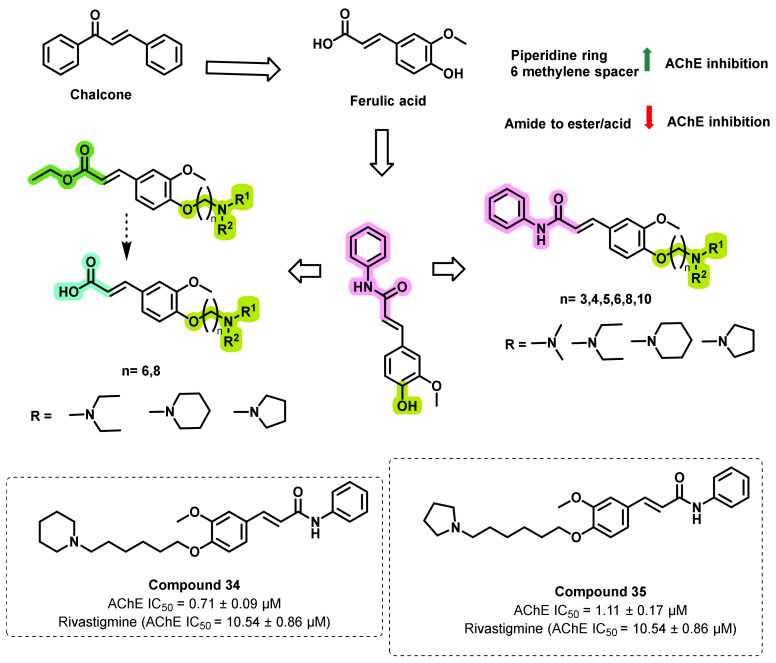
Ferulic acid-inspired chalcone derivatives.

Liu et al., reported the synthesis of a series of novel ferulic acid amide derivatives with tertiary amine side chains and evaluated the AChE and BChE inhibitory potential (Figure 32) [67]. The variation of the spacer linking the ferulic acid scaffold and terminal amine groups dramatically influenced the AChE inhibition properties. The compounds with a six methylene spacer showed better inhibition activity against AChE. Most of the time, the piperidine ring containing amines exerted the potential AChE inhibition. However, for the case of eight or ten spacers, dimethyl or diethyl functionalities exerted the potential AChE activities. Among new synthesized compounds, nine compounds showed better inhibitory activity (IC_50_ = 0.71–8.40 µM) than the control drug rivastigmine (IC_50_ = 10.54 µM). The most promising compounds, 34 (IC_50_: 0.71 ± 0.09 µM) and **35** (IC_50_: 1.11 ± 0.17 µM), possessed 15-fold and 9-fold more inhibitory activity, respectively, against AChE than that of rivastigmine. Moreover, compound 34 showed the highest selectivity for AChE over BChE (ratio: 18:3). The replacement of benzamide group by ethyl ester/acid functionalities resulted in a decrease in the AChE inhibition properties. An enzyme kinetic and molecular docking study (PDB ID: 1EVE) revealed that compound *34* presented a mixed-type inhibition against AChE. Further, the synthesized compounds showed a good lipophilicity (logP value in the range of 1.29 to 1.98) [67].

### 4.2. Chalcone Derivatives with Structural Modifications

Apart from the incorporation of various pharmacophoric features into a single chemical entity, numerous structural modification efforts have been performed for the discovery of potent ChE inhibitors. This includes the alteration of α, β unsaturated carbonyl functional group into rigid analogs, generation of two/three chalcone-coupled moieties via various linkers, structural expansion/reduction of A and/or B rings, open chalcones to rigid chalcones, and attachment of denser aromatic functionalities to chalcone via various functionalities. These kinds of structural modifications result in varied inhibitory activities.

**Figure 33:**
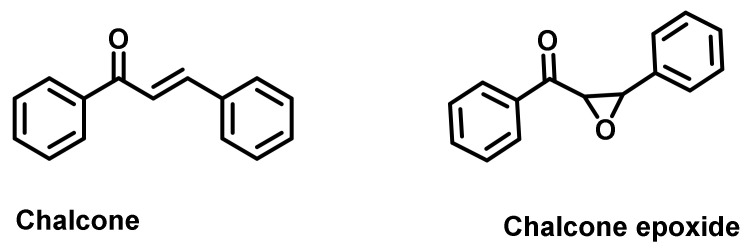
Chemical structure of chalcone and chalcone epoxide.

Stellenboom examined the AChE inhibitory effects of chalcone and its epoxidated derivative (chalcone epoxide) (Figure 33) [68]. Epoxides of the chalcone were prepared by the Weitz–Scheffer epoxidation. Both of the analogs resulted in low micromolar activities against ChEs (chalcone: 1.74 μM (AChE) and 1.43 μM (BChE); chalcone epoxide: 1.91 μM (AChE), and 1.11 μM (BChE)). Rivastigmine was used as a positive control (0.06 and 0.01 μM for AChE and BChE, respectively) [68].

**Figure 34:**
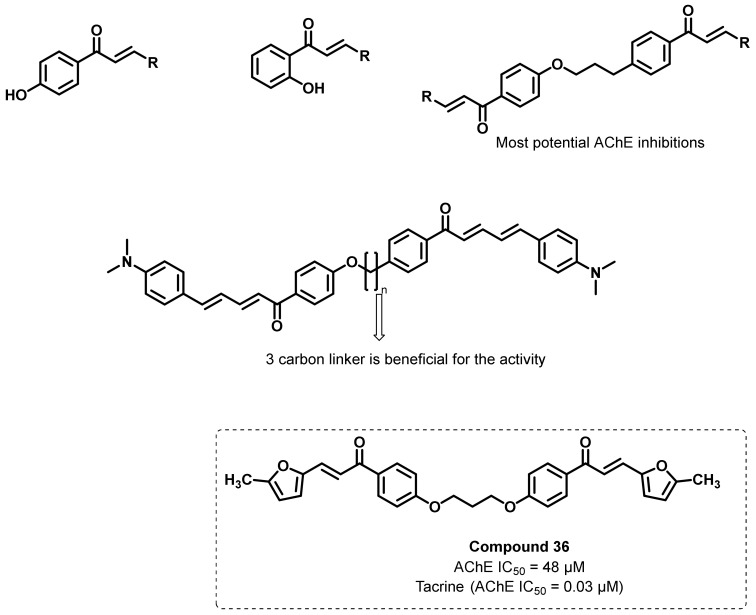
Chemical structure of bis-etherified bis-chalcone.

Liargkova et al., synthesized numerous 2-/4-hydroxy chalcone and bis etherified bis-chalcone analogs and evaluated the ChEs inhibitory potential (Figure 34) [69]. Chalcone analogs were synthesized by the Claisen–Schmidt reaction, while the Williamson reaction was used for the synthesis of dimerization. Numerous aromatic aldehydes containing thiofuran, furan, and quinoline were used as starting materials for understanding the effects of the aromatic functionalities in ChE inhibition. All of the bis-chalcone analogs exerted higher inhibitory potential than the chalcone analogs. Further, the linker space between the ether-linked bis-chalcone was investigated (2 to 8 carbons), wherein three-carbon was found to be the most active ChE inhibitory potential (compound 36; AChE IC_50_ = 48 μM). It is evident from the logP value (2.41 to 9.44) that the majority of the synthesized analogs can cross the BBB [69].

**Figure 35:**
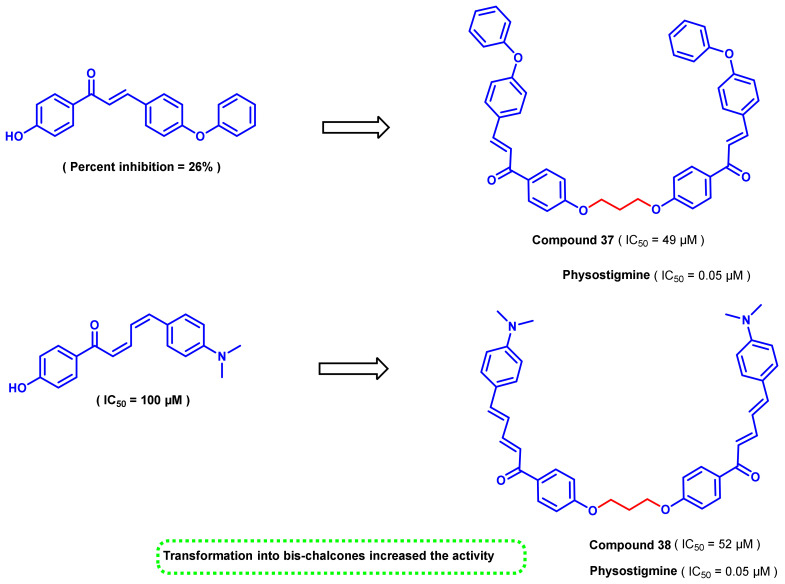
Chemical structures of 4-hydroxy chalcones and bis-chalcone derivatives.

In another study, a series of 4-hydroxy chalcones and bis-chalcones were synthesized and evaluated for their AChE inhibitory activity (Figure 35) [70]. The bis-chalcones consisted of substituted 4-hydroxy chalcones connected via an ether linkage with three carbon linkage (-OCH_2_CH_2_CH_2_O-). The studies indicated that the bis-chalcone analogs (compounds 37 and 38) exhibited more significant inhibition than the simple 4-hydroxy chalcone series. These studies further suggested the importance of bis-analogs for AChE inhibition [70].

**Figure 36:**
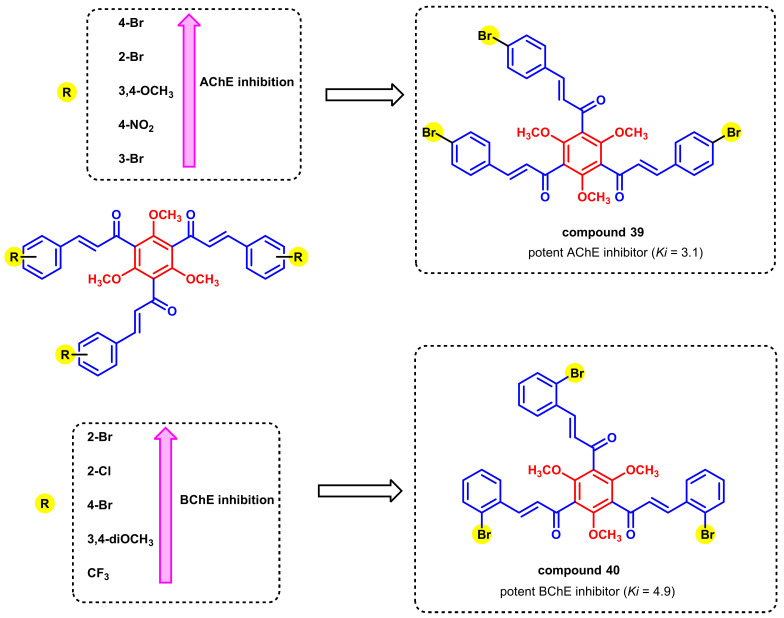
Chemical structures of tris-chalcone derivatives.

The effects of numerous substituted tri-chalcones in AChE inhibition were also reported previously by Burmaoglu et al., (Figure 36) [71]. Three chalcone moieties were connected together by a common trimethoxy-substituted benzene A ring. The -Br-substituted analogs exerted potential ChEs inhibition, wherein compound 39 with 4-bromo substitution on the B ring was the most potent AChE inhibitor (K_i_ = 3.1), and compound 40 with 2-bromo substitution on the B ring was the most potent BChE inhibitor (K_i_ *=* 4.9) [71].

**Figure 37:**
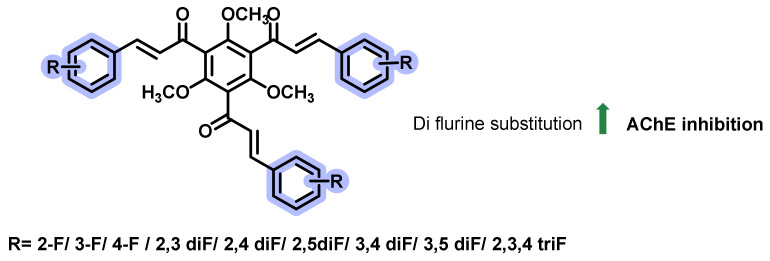
Fluorine-substituted tris-chalcone derivatives.

In another study, Burmaoglu et al., examined the role of novel fluorine-substituted tris-chalcones for their AChE inhibition potential (Figure 37) [72]. The treatment of phloroglucinol with acetic anhydride in the presence of methanesulfonic acid (MSA) resulted in the corresponding acylated derivative. Further *O*-alkylation followed by a base catalyzed reaction with substituted aldehydes resulted in the title analogs. The screened analogs exerted K_i_ values ranging from 1.09 ± 0.20 to 6.84 ± 1.24 nM for AChE, and 85.24 ± 1.71 to 8.35 ± 0.54 nM for BChE. Tacrine was used as a positive control, possessing a K_i_ value of 5.99 ± 1.79 and 2.43 ± 0.92 nM towards AChE and BChE, respectively. Difluorine substitution in the chalcone framework resulted in enhanced activities (AChE, K_i_ = 1.09 to 2.86 nM; BChE, K_i_ = 5.24 to 5.50 nM). The variation in the inhibitory activities w.r.to substituent positions needs to be explored further for the deduction of SAR [72].

**Figure 38:**
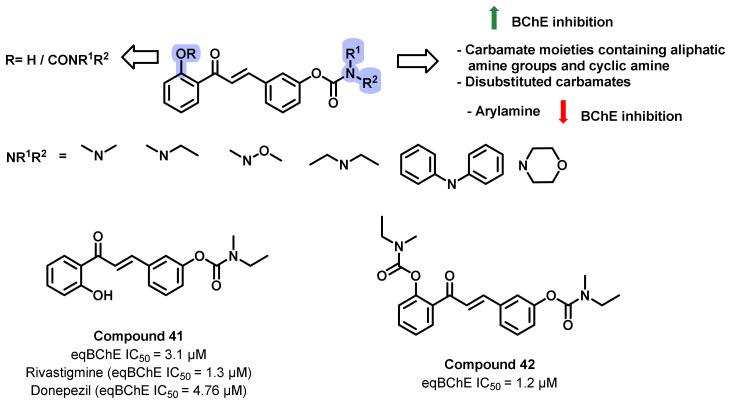
Chemical structures of chalcone-O-carbamate derivatives.

Sang et al., reported the design and synthesis of a series of chalcone-*O*-carbamate derivatives and evaluated their ChE inhibitory potential (Figure 38) [73]. The synthetic schemes were involved in the condensation of 2-hydroxy acetophenone with 3-hydroxybenzaldehyde. The obtained intermediate was treated with *N*,*N*-disubstituted carbamoyl chlorides, resulting in the desired analogs. Carbamate-containing chalcone derivatives exerted weaker AChE inhibition; however, significant inhibition was observed with the BChE inhibition (IC_50_ = 1.2 to 56.2 µM). Rivastigmine was used as a positive control (BChE inhibition: IC_50_ = 1.3 µM). These reports suggested that the incorporation of carbamate does not produce any significant effects on the AChE inhibition. In addition, the carbamate substitution significantly affected the eqBChE inhibitory activity. Aliphatic series *N*,*N*-disubstituted carbamoyl contributed to the eqBChE inhibitory activity, while the aromatic *N*,*N*-disubstituted carbamoyl resulted in a negative effect on eqBChE inhibitory potency. Furthermore, the carbamate moieties at both 2′ and 3′ positions of the chalcone also had significant effects on the eqBChE inhibitory activity. Generally, the disubstituted carbamate derivatives exhibited better eqBChE-inhibitory activity than the monosubstituted carbamate derivatives. Moreover, for the disubstituted carbamate derivatives, the potencies to inhibit eqBChE were in the order *N*-ethyl-*N*-methylamine > *N*-methoxymethylamine > morpholine > *N*,*N* diethylamine > *N*,*N*-dimethylamine > *N*,*N*-diphenylamine. The results revealed that carbamate moieties containing aliphatic amine groups and cyclic amine contributed to the eqBChE-inhibitory activity, but the arylamine produced an adverse effect on eqBChE-inhibitory activity. As for the eeAChE inhibition, the carbamate moieties did not show a significant effect on AChE-inhibitory activity. Therefore, the target compounds were promising selective eqBChE inhibitors, and compounds 41 and 42 were selected for further study. In the in vitro PAMPA BBB assay, compounds 41 and 42 showed a Pe value of 15.67 and 23.17 × 10^−6^ cm/s, indicating their potential for BBB permeation [73].

**Figure 39:**
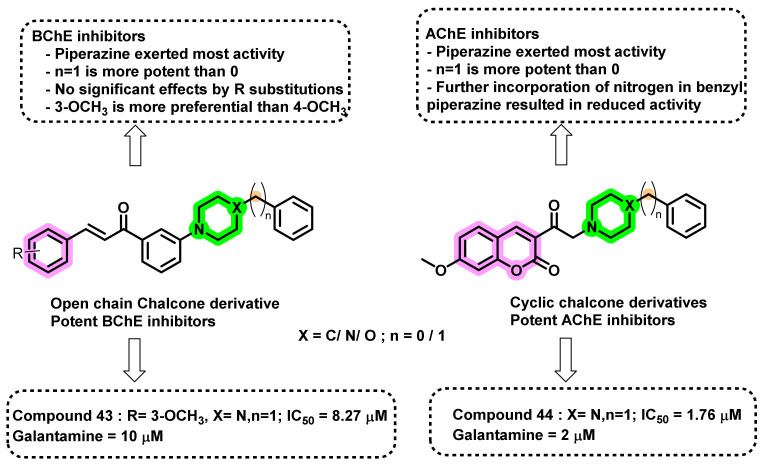
Chemical structures of chalcones reported by Bag et al.

Bag et al., reported the design and synthesis of new multifunctional α,β-unsaturated carbonyl scaffolds and evaluated their ChE inhibition activity (Figure 39) [74]. The design strategy mainly involved the utilization of α,β-unsaturated carbonyl scaffold as open-chain (chalcone) and cyclic forms (coumarin) that were further linked to different amines. Among the screened analogs, the cyclic forms exerted higher ChE inhibition activities than the open chain conformations. Except compound 44, all of the remaining analogs exerted lesser inhibitory potential than the standard drug (galantamine). Compound 44 exhibited higher activity than galantamine, with an IC_50_ of 1.76 µM. In the case of BChE inhibition, the chalcone analogs exerted more activity than the coumarin-type analogs. However, all of the synthesized analogs exerted less activity compared to galantamine at 10 µM. Molecular docking studies highlighted that the coumarin compound 44 was properly oriented in the active site (PDB ID: 1E66). Compound 44 was stabilized by numerous hydrophobic interactions, π-π stacking, and H-bonding. Overall, the study suggested that the cyclic derivatives of α,β-unsaturated carbonyl scaffolds are more potent than the open chain in exerting ChE inhibition. In addition, the chalcones showed strong activity in BChE and fibrillogenesis inhibition only. In contrast, the coumarins performed more consistently, with significant inhibition in all assays, except against BChE, where they were moderate to weak inhibitors. At this point, the coumarin derivatives were limited to one head group and different tail groups possessing aryl-substituted piperidine, piperazine, and morpholine units. Compounds with the two nitrogen-containing piperazine rings performed the best overall. It appeared that the presence of basic nitrogens in the middle ring was advantageous; however, too many nitrogens (e.g., pyrimidinylpiperazine group) resulted in a slight decrease in activity in all assays [74].

**Figure 40:**
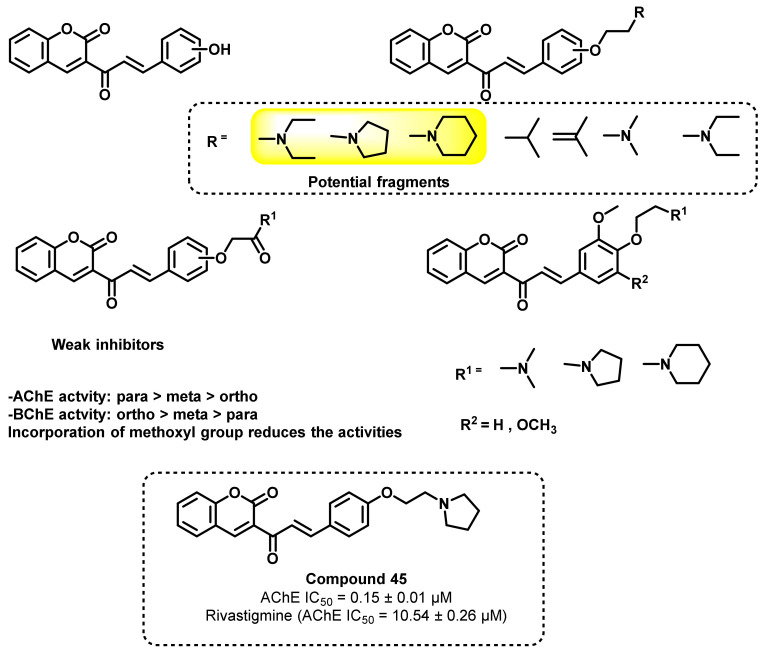
Chemical structures of coumarin–chalcone hybrid analogs.

In another study by Lu Kang et al., a series of coumarin–chalcone hybrid analogs was reported (Figure 40) [75]. The study emphasized the effects of tertiary amine side chains (positions) on AChE inhibitory potential. Para-substituted chalcone fragments exerted higher AChE inhibitory potential over BChE. The similarity in these structures with the choline might be the probable reason for the enhancement of AChE inhibition. Further replacement of the simple amine functionalities to amide, alkyl, or alkenyl groups resulted in an abrogated activity, further highlighting the importance of amine functionalities for ChE inhibition. Compound 45 showed potent inhibitory activity (IC_50_ = 0.15 ± 0.01 μM) with a good selectivity for AChE over BChE (ratio 27:4). In kinetic studies, these analogs exhibited a mixed-type inhibition against AChE. Molecular modeling studies (PDB ID: 1EVE/1P0I) also supported these facts, wherein these analogs interacted with the peripheral active sites (PAS) as well as the catalytic active sites (CAS) [75].

**Figure 41:**
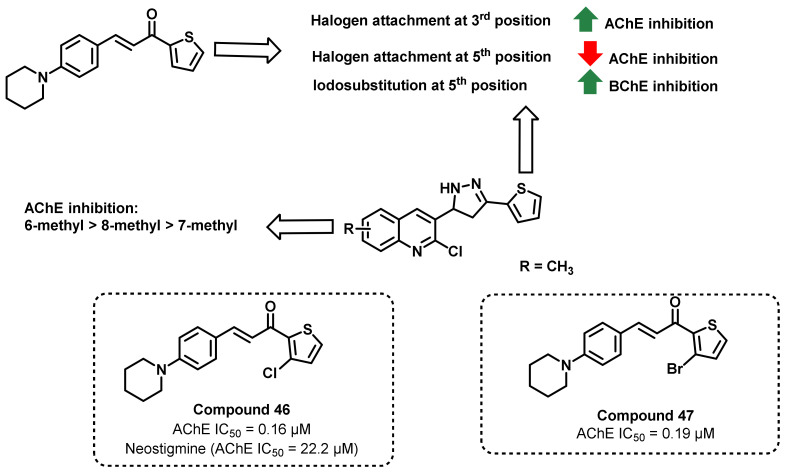
Piperidyl–thienyl and 2-pyrazoline derivatives of chalcone.

Shakil Shah et al., reported a series of piperidyl-thienyl and 2-pyrazoline derivatives of chalcone and evaluated them against AChE and BChE (Figure 41). *N*-Arylation of piperidine with 4-flurobenzaldehyde resulted in the formation of 4-(Piperidine-l-yl) benzaldehyde, which was further condensed with different substituted acetyl thiophene, resulting in the formation of piperidyl–thienyl chalcones. In another set, 2-pyrazoline-quinoline-containing chalcones were prepared by the treatment of 2-chloro-3-formyl quinolines with substituted acetyl thiophenes, followed by the treatment of hydrazine hydrate. The synthesized analogs demonstrated good inhibitory potential against AChE, while poor activity was obtained against BChE. Neostigmine was used as a standard inhibitor (IC_50_ = 22.2 µM). Analogs possessing 3-chlorothiophen-2-yl and 3-bromothiophen-2-yl (Compounds 46 and 47) moiety at the piperidine chalcones exhibited the highest AChE inhibition properties (IC_50_ = 0.16 and 0.19 µM, respectively). In the enzyme inhibition kinetic experiments, piperidyl–thienyl chalcones showed a competitive mode of inhibition, while 2-pyrazoline-quinoline demonstrated a mixed type of enzyme inhibition. A molecular docking study (PDB ID: 1EVE) revealed that potent compounds and co-crystallized ligand had the same binding orientation within the active site of the target enzyme [76].

**Figure 42:**
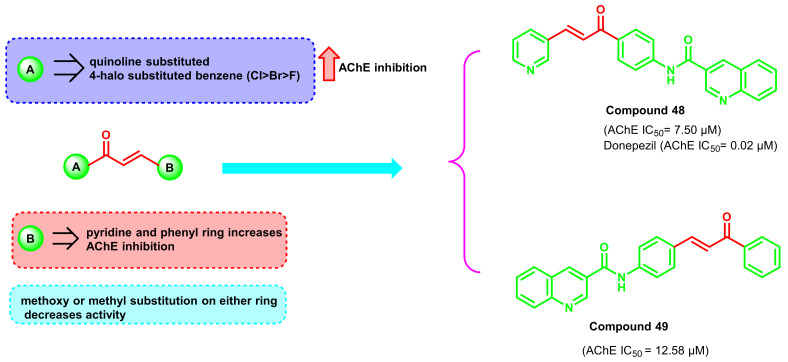
Chemical structures of quinolone-substituted chalcone derivatives.

Conventional chalcone synthesis is usually performed with the Claisen–Schmidt synthesis, which takes around 6 to 72 h. Numerous efforts have been made to develop the chalcone in a faster and more eco-friendly manner. The ultrasound-assisted synthesis strategy is one of the reported methods, wherein the reaction time is reduced to 20 min. Interestingly, the yield of the compounds depends on the nature and type of substituents. A higher substituted chalcone was formed in a higher yield, while an inverse correlation was observed with the electronegativity (Cl > Br > F). A varied amount of ChEs inhibitory potential was revealed by the heterocyclic chalcones (Figure 42) [77]. Substituted quinoline derivatives (especially with halogen) on the A ring exerted higher ChE inhibitory profiles, while the pyridinyl and phenyl rings were found to be promising in the B counterparts. Morpholine substitutions at the B ring parts resulted in ChE inhibition. Further, methoxy and methyl substitution generally decreased the activity, as in the case with methyl substitution. The most potent series of compounds were the quinoline chalcones with compound 48 (IC_50_ = 7.50 μM) and compound 49 (IC_50_ = 12.58 μM) [77].

**Figure 43:**
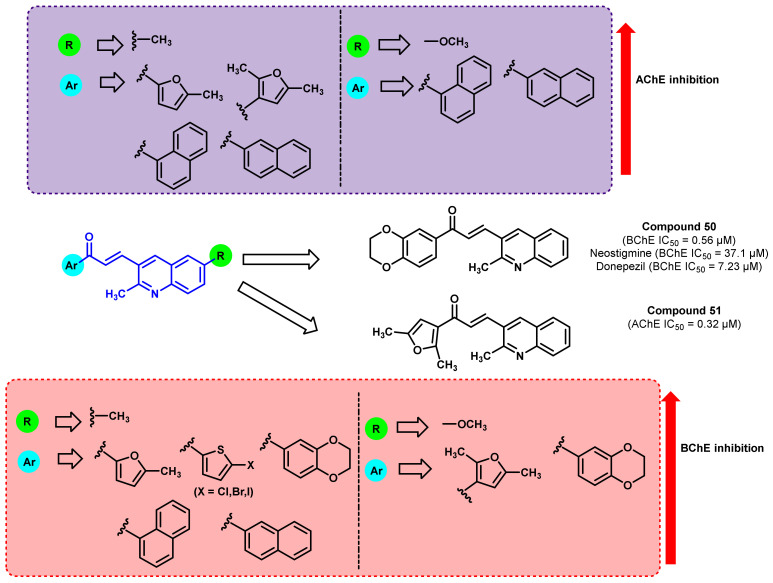
Structural modification of quinolone-substituted chalcone derivatives.

The effects of denser hydrocarbons such as quinoline associated with chalcones were also screened for their anticholinesterase activity (Figure 43) [78]. Numerous aryl and heteroaryl rings were selected as the A ring, while 2,6-dimethyl quinoline was selected for the synthesis of one series and 6-methoxy-2-methyl quinoline was used as another set of the library. Interestingly, these analogs revealed a potent BChE nature over AChE inhibition. Compound 50 exerted the potential BChE inhibition (IC_50_ = 0.56 μM). The potential activity was assumed mainly due to the higher electronegativity of the attached 1,4 dioxane ring to ring A. Further, the dioxane-substituted compound with 6-methoxy substitution on the quinoline ring showed a decrease in activity, whereas the same compound with 6-methyl substitution showed significant AChE inhibition, highlighting the importance of the methyl group. Compound 51 exhibited significant AChE inhibition, which might be due to the high influence of the dimethyl furan ring as it is present in the A ring (IC_50_ = 0.32 μM). Based on the total polar surface area (TPSA) descriptor, it is suggested that these analogs (TPSA < 40) possessed greater BBB permeation [78].

**Figure 44:**
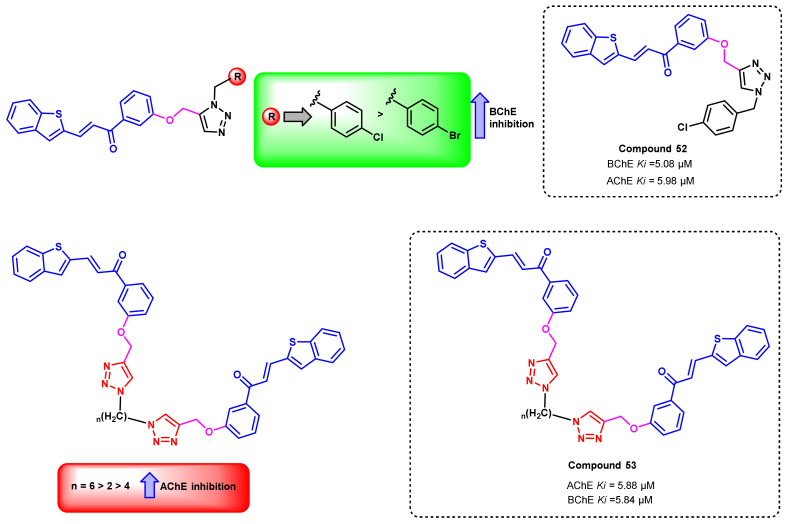
Chemical structures of triazole-chalcone derivatives.

In another report, a hybrid of 1,2,3-triazole and chalcones was designed and synthesized, wherein benzothiophene was incorporated into the B ring counterparts (Figure 44) [79]. Upon in vitro AChE inhibition evaluations, these analogs exerted moderate to good potentials (5.88–11.13 μM). Furthermore, bis-chalcone triazole derivatives were also developed, wherein 6 carbon linkers (methylene) showed potent AChE inhibition (compound 53) (AChE, K_i_ = 5.88 μM). However, 4-chlorobenzyl 1,2,3-triazole substituted chalcones exerted the potent BChE inhibition (compound 52) (BChE, K_i_ *=* 5.08 μM) [79].

**Figure 45:**
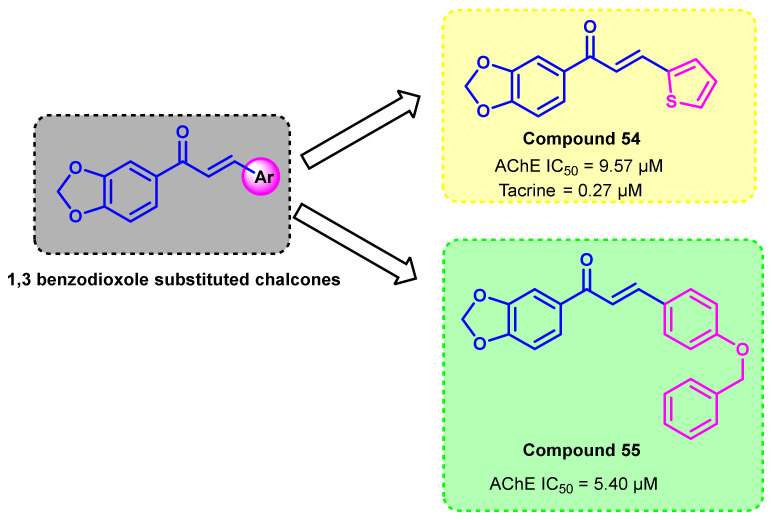
Chemical structures of benzodioxole chalcone derivatives.

1,3-Benzodioxole chalcones were evaluated for their multi-targeting potential (Figure 45) [80]. Thiophene and benzyloxy benzene-containing chalcones (compounds 54 and 55) showed promising effects for AChE inhibition. A clear-cut SAR could not be derived, but it highlighted the importance of the 1,3-benzodioxole ring as a replacement of the A phenyl ring in chalcones and its potency towards the multi-targeting role. In silico analysis revealed that these compounds possess higher BBB permeation properties [80].

**Figure 46:**
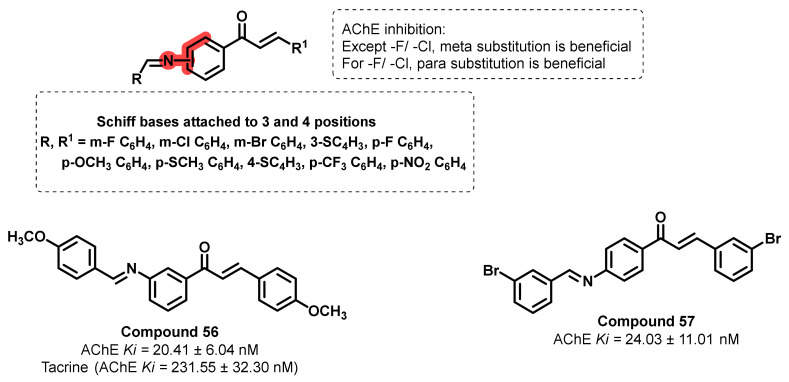
Chemical structures of Schiff bases-appended chalcone derivatives.

Koçyiğit et al., disclosed the design and synthesis of a series of new Schiff bases-appended chalcone derivatives (in the meta and para positions of the chalcones scaffold) (Figure 46) [81]. Initially, the reaction of various aromatic aldehydes with the acetyl part of aminoacetophenones resulted in the formation of chalcone skeletons. Further treatment of these analogs with various aldehydes afforded the chalcones with Schiff bases. All chalcone derivatives containing Schiff bases exhibited significantly higher AChE inhibitory activity (IC_50_ = 67.33–150.98 nM) than tacrine (IC_50_ = 436.00 nM). Further, the K_i_ values were in the range of 20.41 ± 6.04 to 125.94 ± 23.88 nM. The most active compounds, 56 and 57, showed K_i_ values of 20.41 ± 6.04 and 24.03 ± 11.01 nM, respectively. Except for -Cl- and -F-substituted analogs, meta-substituted Schiff bases analogs exerted more potential activity than the para substitutions. On the contrary, para-position substitutions of Schiff bases with -Cl and -F substitutions resulted in the potential AChE inhibition potential (IC_50_ = 72.77 and 67.33 nM, respectively). Overall, these reports suggested the effects of substituents effects in AChE inhibition [81].

**Figure 47:**
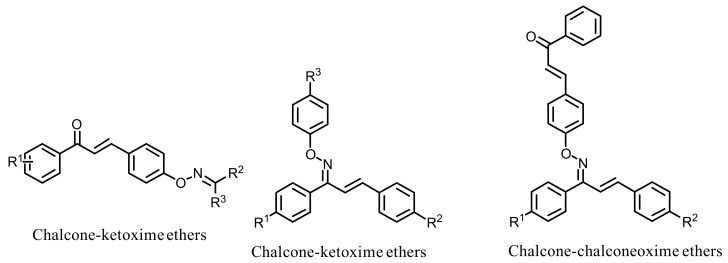
Chemical structures of chalcone oxime ether derivatives.

One-pot palladium catalyzed C-O cross-coupling of activated aryl bromides, ketoximes, and chalcone oximes resulted in the formation of chalcone oxime ethers and their ChE inhibitory potential was disclosed by Oh et al., (Figure 47) [82]. The synthesized derivatives included the classes of chalcone-ketoxime ethers, aryl chalcone oxime ethers, and chalcone-chalconeoxime ethers. Amongst the synthesized analogs, chalcone-chalconeoxime ethers demonstrated the highest AChE inhibitory potential. However, these analogs exerted weaker inhibitory potential (4.39 to > 40 µM) than that of the standard drug (Tacrine, IC_50_ = 0.20 µM) [82].

## 5. Conclusions and Perspectives

AD is a progressive neurodegenerative disorder and affects the entire globe. Though numerous medications are available for the management of these conditions, these medications lack certain kinds of efficacy parameters. Amongst the numerous scaffolds explored for the development of novel agents for AD, chalcone is one of the privileged scaffolds, possessing diverse biological functions. Numerous reports available on the ChEI potential of chalcone-derived agents are provided in the present article. The development of chalcone-based inhibitors mainly relies on the hybridization approach, wherein numerous chemical functionalities are incorporated into the single chemical entity. Various kinds of structural modification approaches have been explored for the identification of chalcone-inspired analogs. It includes the evaluation of various open or closed forms of numerous amine, the replacement of simple hydroxyl functionalities to ether derivatives, the replacement of simple aromatic functionalities to the denser/lighter heterocyclic rings, and the bis- or tris-type of chalcone derivatives. Apart from that, combinations of the USFDA-approved chalcones are also reported. The majority of these results are also supported by molecular modelling approaches as well.

The substitution of various functional groups resulted in altered ChE inhibitory properties. For instance, EDG was reported to have enhanced AChE inhibition, while the opposite effect was observed with EWG. The presence of multiple functionalities, derived from -*O* and -*N*, exerted an overall enhancement in AChE inhibitory properties. The ring constriction of chalcone (especially the B ring) results in decreased potential towards ChEs. The modification of simple -NH_2_ to a cyclic form was also reported by numerous researchers. It is interesting to note that, except for cyclic imides, all of the reported cyclic amide derivatives exerted maximum ChE inhibitory potential when a EWG is present at ring B. Interchanging -NH_2_ to sulphonamide derivatives revealed an increase in BChE inhibitory properties.

The conversion of -OH functionalities to ester and ether has also been explored by various researchers, wherein ethers with amino terminal functionalities exerted maximum ChE inhibition. Depending on the alkyl chain length, the ChE inhibitory properties also varied. For instance, a four-carbon spacer exerted potential activity in cyclic amine functionalities, while a five-carbon spacer was beneficial for simple alkyl amines. Further, the effect of EWG on the B ring under these conditions was also evaluated. Meta-substituted analogs exerted weaker activity than the ortho and para substitutions. Furthermore, the ortho position was beneficial for ether derivatives with a simple alkyl amine derivative, while the para-position substitution was beneficial for ethers with a cyclic amine-type terminal group. The attachment of an alkyl chain through an ether linkage via both of the aromatic rings was also explored. A six-carbon spacer was found to be the most beneficial spacer for these kinds of analogs. Structural modifications of amino terminals with other functional groups, such as the addition of carbamates, benzyl piperidine, and tetrahydroisoquinoline derivatives, have also been reported. In the case of carbamates attached to the chalcone scaffold via ether linkage, three- to seven-carbon spacers demonstrated the optimum desired activity. In the case of the benzyl piperidine modification, substitution with EDG groups resulted in enhanced ChE inhibitory potential, while in the case of tetrahydroisoquinoline, attachment at the B ring resulted in BChE inhibition and attachment at the A ring resulted in enhanced AChE inhibitory potential. Similar to the previous cases, EDG groups revealed an enhancement in the activity, while EWG resulted in decreased ChE inhibitory potential.

In another approach, clinically used drugs appending chalcone derivatives were also explored for the identification of potential ChE inhibitors. The *N*-Benzyl pyridinium motif (inspired from donepezil) with benzofuran-containing chalcones, donepezil–chalcone hybrid analogs, carbamates from rivastigmine to chalcone, and flurbiprofen–chalcone analogs are the few representative examples for novel agents conceived from the USFDA-approved drugs modifications.

**Figure 48:**
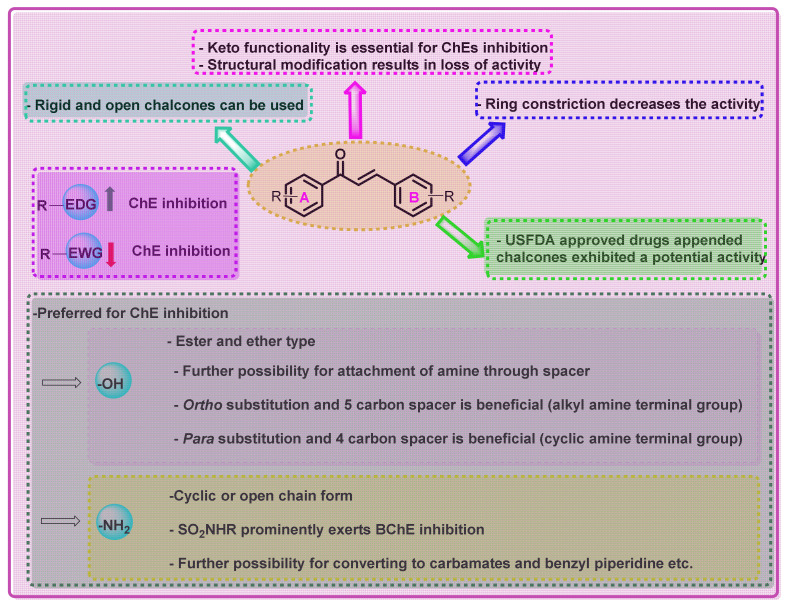
Summary of SAR points in ChE inhibition.

The structural modification of the chalcone scaffold has also been explored for the development of ChE inhibitors, which resulted in a varied effect. For instance, unsaturated ketone into saturated derivatives or pyrazoline and chalcone to chalcone epoxide resulted in the reduction of ChE inhibitory properties. Further, the conversion of rotatable chalcone to rigid chalcone resulted in an enhancement of the ChEs inhibitory profile. It is also interesting to note that the bis- and tris-type of chalcone derivatives exerted enhanced ChE inhibition. The SAR points in this review are summarized in Figure 48.

Overall, the information compiled in this review will provide a detailed SAR of various functional groups, which are associated with the chalcone scaffolds. The authors expect that this detailed information will encourage the medicinal chemist fraternity to explore chalcones as potential therapeutic agents for AD.

## Data Availability

Not applicable.
